# Crystal structure of *Grimontia hollisae* collagenase provides insights into its novel substrate specificity toward collagen

**DOI:** 10.1016/j.jbc.2022.102109

**Published:** 2022-06-06

**Authors:** Takeaki Ikeuchi, Mizuki Yasumoto, Teisuke Takita, Keisuke Tanaka, Masashi Kusubata, Osamu Hayashida, Shunji Hattori, Kimihiko Mizutani, Bunzo Mikami, Kiyoshi Yasukawa

**Affiliations:** 1 Division of Food Science and Biotechnology, Graduate School of Agriculture, Kyoto University, Sakyo-ku, Kyoto, Japan; 2 Nippi Research Institute of Biomatrix, Toride, Ibaraki, Japan; 3 Division of Applied Life Sciences, Graduate School of Agriculture, Kyoto University, Uji, Kyoto, Japan; 4 Research Institute for Sustainable Humanosphere, Kyoto University, Uji, Kyoto, Japan; 5 Institute of Advanced Energy, Kyoto University, Uji, Kyoto, Japan

**Keywords:** collagenase, *Grimontia hollisae*, crystal structure, collagen, Gly-Pro-Hyp, Act1 and Act2, the α-helices 5−9 and 10−19, respectively, in the activator domain of Ghcol, C1 and C2, the upper-half and lower-half subdomains in the peptidase domain of Ghcol, CAT, catalytic domain, ColG, *Clostridium histolyticum* class I collagenase, ColH, *Clostridium histolyticum* class II collagenase, ColT, *Clostridium tetani* collagenase, FALGPA, *N*-[3-(2-furyl)acryloyl]-Leu-Gly-Pro-Ala, Ghcol, collagenase from *Grimontia hollisae* strain 1706B, HPX, hemopexin-like domain, Hyp, hydroxyproline, MMP, matrix metalloproteinase, MOCAc-KPLGL(Dpa)-AR, (7-methoxycoumarin-4-yl)acetyl-Lys-Pro-Leu-Gly-Leu-[*N*^3^-(2,4-dinitrophenyl)-2,3-diaminopropionyl]-Ala-Arg, PDB, Protein Data Bank, THP, triple-helical peptide, VhaC, *Vibrio harveyi* collagenase

## Abstract

Collagenase from the gram-negative bacterium *Grimontia hollisae* strain 1706B (Ghcol) degrades collagen more efficiently even than clostridial collagenase, the most widely used industrial collagenase. However, the structural determinants facilitating this efficiency are unclear. Here, we report the crystal structures of ligand-free and Gly-Pro-hydroxyproline (Hyp)-complexed Ghcol at 2.2 and 2.4 Å resolution, respectively. These structures revealed that the activator and peptidase domains in Ghcol form a saddle-shaped structure with one zinc ion and four calcium ions. In addition, the activator domain comprises two homologous subdomains, whereas zinc-bound water was observed in the ligand-free Ghcol. In the ligand-complexed Ghcol, we found two Gly-Pro-Hyp molecules, each bind at the active site and at two surfaces on the duplicate subdomains of the activator domain facing the active site, and the nucleophilic water is replaced by the carboxyl oxygen of Hyp at the P1 position. Furthermore, all Gly-Pro-Hyp molecules bound to Ghcol have almost the same conformation as Pro-Pro-Gly motif in model collagen (Pro-Pro-Gly)_10_, suggesting these three sites contribute to the unwinding of the collagen triple helix. A comparison of activities revealed that Ghcol exhibits broader substrate specificity than clostridial collagenase at the P2 and P2′ positions, which may be attributed to the larger space available for substrate binding at the S2 and S2′ sites in Ghcol. Analysis of variants of three active-site Tyr residues revealed that mutation of Tyr564 affected catalysis, whereas mutation of Tyr476 or Tyr555 affected substrate recognition. These results provide insights into the substrate specificity and mechanism of *G. hollisae* collagenase.

Collagen is the most abundant protein in mammals and has a triple-helical structure, with Gly-Pro-hydroxyproline (Hyp) tripeptide as the basic unit. Collagenase (Enzyme Commission number: 3.4.24.3) cleaves the triple-helical region of collagen under physiological conditions, catalyzing the hydrolysis of peptide bonds with glycine residues at the P1 position (1). Bacterial collagenases are zinc metalloproteinases containing the zinc-binding motif sequence, HEXXH, in their active sites (2). Most bacterial collagenases belong to the M9 MEROPS peptidase family (3), with clostridial collagenase being the most well characterized among them (4, 5, 6, 7, 8, 9, 10). Clostridial collagenase is used industrially as a reagent to isolate pancreatic islet cells for transplantation in diabetes (11). It also finds clinical application in the degradation of native collagen in the fingers of patients with Dupuytren’s contracture (12). In contrast, some bacterial collagenases are important virulence factors in various pathogenic diseases (13, 14, 15, 16, 17). For example, a collagenase from *Flavobacterium psychrophilum* characterized in our previous study (17) causes cold-water disease in ayu fish (*Plecoglossus altivelis*) (18).

The gram-negative bacterial genus *Grimontia* was first described as *Vibrio hollisae* in 1982 (16). *Grimontia hollisae* strain 1706B was isolated from seashore sand collected from the Shin-Kiba coast of Tokyo, Japan (19). It produces the collagenase Ghcol, which consists of 767 amino acid residues with a single catalytic domain containing the zinc-binding motif H^492^EYVH^496^ (Fig. S1) (19). Recombinant Ghcol produced using the *Brevibacillus chosinensis* expression system has been shown to degrade collagen more efficiently than clostridial collagenase (19, 20, 21, 22, 23), thus exhibiting potential for industrial application.

To expand the industrial utility of collagenase, a better understanding of the mechanism by which collagenase cleaves the triple-helical region of collagen is required. However, only limited information is available regarding the crystal structure of clostridial collagenase (8, 9) and *Vibrio* collagenase VhaC (24). Here, we report the crystal structures of ligand-free Ghcol at 2.2 Å resolution and Ghcol complexed with Gly-Pro-Hyp at 2.4 Å resolution. Furthermore, we compared the activities and substrate specificities of Ghcol and clostridial collagenase and examined the role of three Tyr residues in the active site of Ghcol on its catalytic activity and substrate specificity.

## Results and discussion

### Determination of Ghcol structure

In our previous study, a Ghcol protein (Ala88–Gln767) with the 30-amino acid Sec signal peptide (Met1–Ala30) added at the N terminus for extracellular secretion was expressed using the *B. chosinensis* expression system (Fig. S2*A*) and purified from the culture supernatant (23). The purified protein preparation exhibited 62- and 74-kDa bands on SDS-PAGE under reducing conditions, suggesting that the C-terminal region (12 kDa) of the 74-kDa Ghcol was degraded during the purification steps (23). To obtain a homogeneous preparation, we expressed the collagenase module (Ala88–Thr646) of Ghcol with the Sec signal peptide using the *B. chosinensis* expression system (Fig. S2*B*) and purified it from the supernatant. This purified Ghcol preparation was homogenous, exhibiting a single 62-kDa band on reducing SDS-PAGE (Fig. S3). Crystals of ligand-free Ghcol were obtained (Fig. S4). Crystals of Ghcol complexed with Gly-Pro-Hyp were prepared by soaking the crystals of ligand-free Ghcol in a reservoir solution containing Gly-Pro-Hyp. Table 1 summarizes the data collection and structure statistics. The space groups of ligand-free and ligand-complexed Ghcol structures are *C*2 and *P*2_1_, respectively. Both crystals contain two molecules (chains A and B) in the asymmetric unit of the cell. The structures of ligand-free and ligand-complexed Ghcol (Ala88–Gly622) were refined at 2.2 and 2.4 Å resolution with *R*/*R*_free_ of 0.186/0.227 and 0.206/0.255, respectively.

**Table 1:** Data collection and refinement statistics

Ghcol	Ligand-free Ghcol	Ghcol complexed with Gly-Pro-Hyp
A. Diffraction data		
X-ray source	SPring-8/BL26B1	SPring-8/BL26B1
Detector	DECTRIS EIGER 4M	DECTRIS EIGER 4M
Wavelength (Å)	1.0	1.0
Resolution range (Å)	50.00–2.19 (2.32–2.19)	50.00–2.39 (2.54–2.39)
Space group	*C2*	*P*2_1_
Unit cell parameters		
a, b, c (Å) β (°)	182.94, 75.12, 135.15, 128.19	72.97, 75.55, 130.55, 103.06
Unique reflections	72,941 (11,564)	54,059 (8681)
Multiplicity	3.54 (3.61)	3.71 (3.84)
Completeness (%)	98.0 (97.2)	98.5 (98.3)
Mean *I*/σ(*I*)	13.3 (2.05)	12.38 (2.34)
Wilson *B*-factor (Å^2^)	44.27	47.75
*R*_merge_ (%)	7.5 (75.0)	9.3 (72.7)
*R*_meas_ (%)	8.8 (88.0)	10.9 (84.4)
CC_1/2_ (%)	99.8 (89.8)	99.7 (78.7)
B. Refinement statistics		
Resolution range used refinement	44.5–2.19 (2.22–2.19)	45.4–2.39 (2.44–2.39)
*R*_work_ (%)	18.6 (34.7)	20.6 (34.3)
*R*_free_ (%)	22.7 (37.0)	25.5 (39.6)
Number of residues		
Protein	533 (90–622) × 2	533 (90–622) × 2
Zn/Ca/1PE/PGE/PEG/EDO/water	2/8/1/2/4/24/477	2/8/0/0/0/5/157
Peptide (Gly-Pro-Hyp)	—	3 × 12
RMSD, bond lengths (Å)	0.007	0.008
RMSD, bond angles (°)	0.820	0.946
Ramachandran favored (%)	98.8	97.1
Ramachandran outliers (%)	0.19	0.19
Rotamer outliers (%)	0.57	2.29
Clash score	3.41	4.05
PDB ID	7WSS	7XEB

### Overall structure of Ghcol

**Figure 1:**
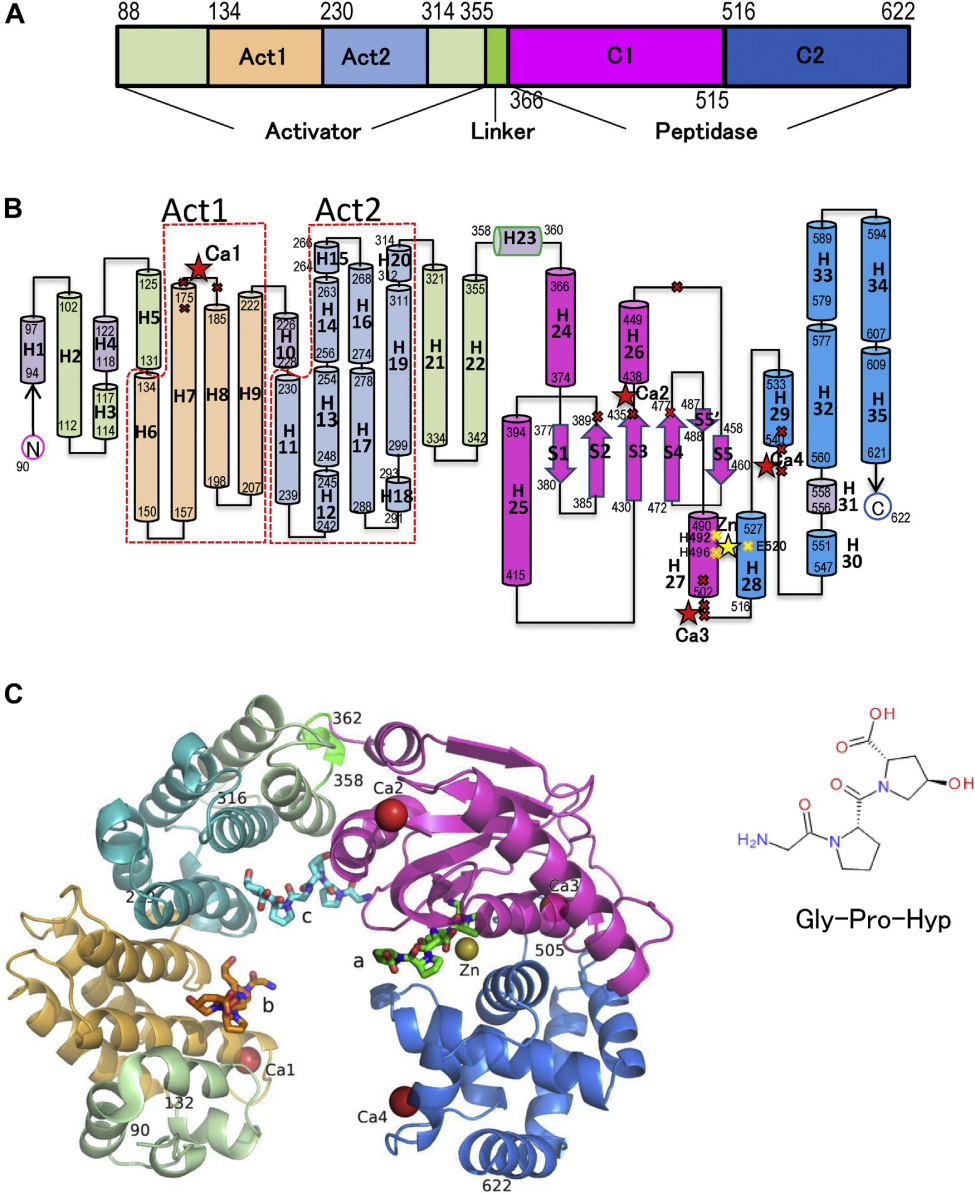
**Overall structure of Ghcol.** Activator domain (Ala88–Tyr355, *silver*, *gold*, and *light blue*), linker (Ala356–Gly365, *green*), upper-half peptidase subdomain (C1: Phe366–Val515, *magenta*), and lower-half peptidase subdomain (C2: Val516–Gly622, *blue*) are shown. The number indicates that of amino acid residues. *A*, domain organization. *B*, topology diagram. The α-helix is shown as a *column*. The β-strand is shown as an *arrow*. The catalytic zinc ion is shown as a *yellow star*. The amino acid residue to which calcium ion (Ca1–4) binds is shown as a *red cross*. *C*, crystallographic structure of Gly-Pro-Hyp-complexed Ghcol. The catalytic zinc ion is shown as a *yellow sphere*. The calcium ions are shown as a *pink sphere* or a *red sphere*. The bound peptides are shown in *stick* with *green* for a in the active site and *orange* for b in the activator domain 1 (Act1) and *cyan* for c in the activator domain 2 (Act2). Ghcol, collagenase from *Grimontia hollisae* strain 1706B.

Figure 1 shows the overall structure of the collagenase module of Ghcol. The structure is divided into the activator domain (Ala88–Tyr355), linker (Ala356–Gly365), and peptidase domain (Phe366–Gly622). The peptidase domain is further divided into two subdomains, namely the upper half (C1: Phe366–Val515) and lower half (C2: Val516–Gly622) (Fig. 1*A*). Figure 1*B* shows topology of the activator and peptidase domains. The activator domain contains 21 α-helices. The α-helices 5–9 (Act1) and 10–19 (Act2) exhibit similar topologies. The peptidase domain comprises the upper-half and lower-half subdomains. In the peptidase domain, the upper-half subdomain contains four α-helices and six β-strands, including the zinc-binding motif H^492^EYVH^496^, whereas the lower-half subdomain contains eight α-helices and no β-strands. Figure 1*C* shows the crystal structure of the collagenase module of Gly-Pro-Hyp-bound Ghcol. The activator and peptidase domains exhibit a saddle-shaped structure with one zinc and four calcium ions. Unexpectedly, Ghcol binds to two peptides at each of three binding sites, which are present in the active site (a) and in the activator subdomains, Act1 (b) and Act2 (c). The sites are separated by a distance of 15 to 27 Å. Each two peptides in the three binding sites extend in the same direction.

**Figure 2:**
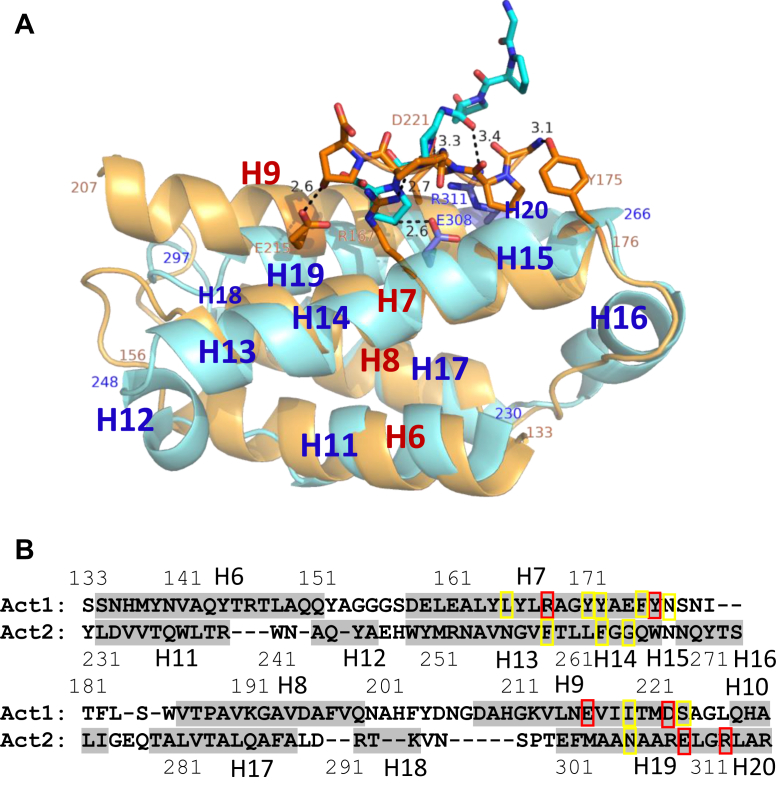
**Comparison of the activator domains 1 and 2 (Act1 and Act2).***A*, crystallographic structure. Gly-Pro-Hyp-bound Act1 (Thr125–Asn223) and Act2 (Gly224–Asp315) are shown in *light orange* and *light blue*, respectively, after superposition. Side chains of the residues (Arg167, Tyr175, Glu215, Asp221, Glu308, and Arg311) with hydrogen bond and C-C contact are shown in *blue stick*. The number indicates the distance (Å) between the atoms. *B*, structure sequence alignment after superposition by COOT. The RMSD for 152 C-alpha pair is calculated to be 3.3 Å. *Red box* indicates the residues with hydrogen bond and C–C contact. *Yellow box* indicates the residues with only C–C contact. The helix region is drawn in *gray color*.

A comparison of tertiary structures of Act1 and Act2 (Fig. 2*A*) revealed that they are similar with an RMSD of 3.3 Å for 152 pair C-alpha atoms. However, Act1 and Act2 do not share a significant sequence homology (Fig. 2*B*). We speculate that in evolution, one of the two subdomains were generated from the other subdomain by gene duplication, and that the amino acid sequences have changed extensively without changing the tertiary structure.

The superposition of the two molecules in the asymmetric unit of ligand-free Ghcol and Gly-Pro-Hyp-bound Ghcol shows RMSD of 0.38 and 0.31 Å for 535 and 533 pair C-alpha atoms, respectively. It becomes smaller to 0.16 to 0.21 Å when each domains are compared (Table 2). The RMSD of each domain between ligand-free and Gly-Pro-Hyp-bound enzymes is also low (0.16–0.23 Å, Table 2), suggesting that almost no conformation change occurred by the binding of Gly-Pro-Hyp. The structure figures in this article were prepared for chain A both in the ligand-free and Gly-Pro-Hyp-bound Ghcol.

**Table 2:** RMSD values between Ghcol (PDB: 7WSS) and other bacterial collagenases

Protein	Activator domain	Peptidase domain	C1 domain	C2 domain
Chain A *versus* B (PDB ID: 7WSS)	0.18 (267)	0.18 (257)	0.16 (150)	0.19 (107)
Chain A *versus* B				
Gly-Pro-Hyp-bound (PDB ID: 7XEB)	0.20 (265)	0.21 (257)	0.15 (150)	0.20 (107)
Gly-Pro-Hyp-bound (PDB ID: 7XEB A)	0.23 (266)	0.20 (257)	0.16 (150)	0.20 (107)
ColG (PDB ID: 2Y50)	2.38 (233)	2.06 (224)	1.78 (133)	1.81 (101)
ColH (PDB ID: 4AR1)	—	1.88 (223)	1.72 (121)	1.64 (105)
ColT (PDB ID: 4AR9 A)	—	1.89 (233)	1.64 (133)	1.56 (101)
VhaC (PDB ID: 7ESI)	0.61 (262)	1.38 (255)	1.11 (149)	0.35 (107)

RMSD values (Å) of C-alpha atoms and compared residue numbers in parentheses are calculated by COOT after superposition of chain A in ligand-free Ghcol (PDB ID: 7WSS) to the proteins of the *left column*. C1 and C2 designate the upper-half and the lower-half subdomains, respectively, in the peptidase domain.

### Structures of Gly-Pro-Hyp binding sites of Ghcol

**Figure 3:**
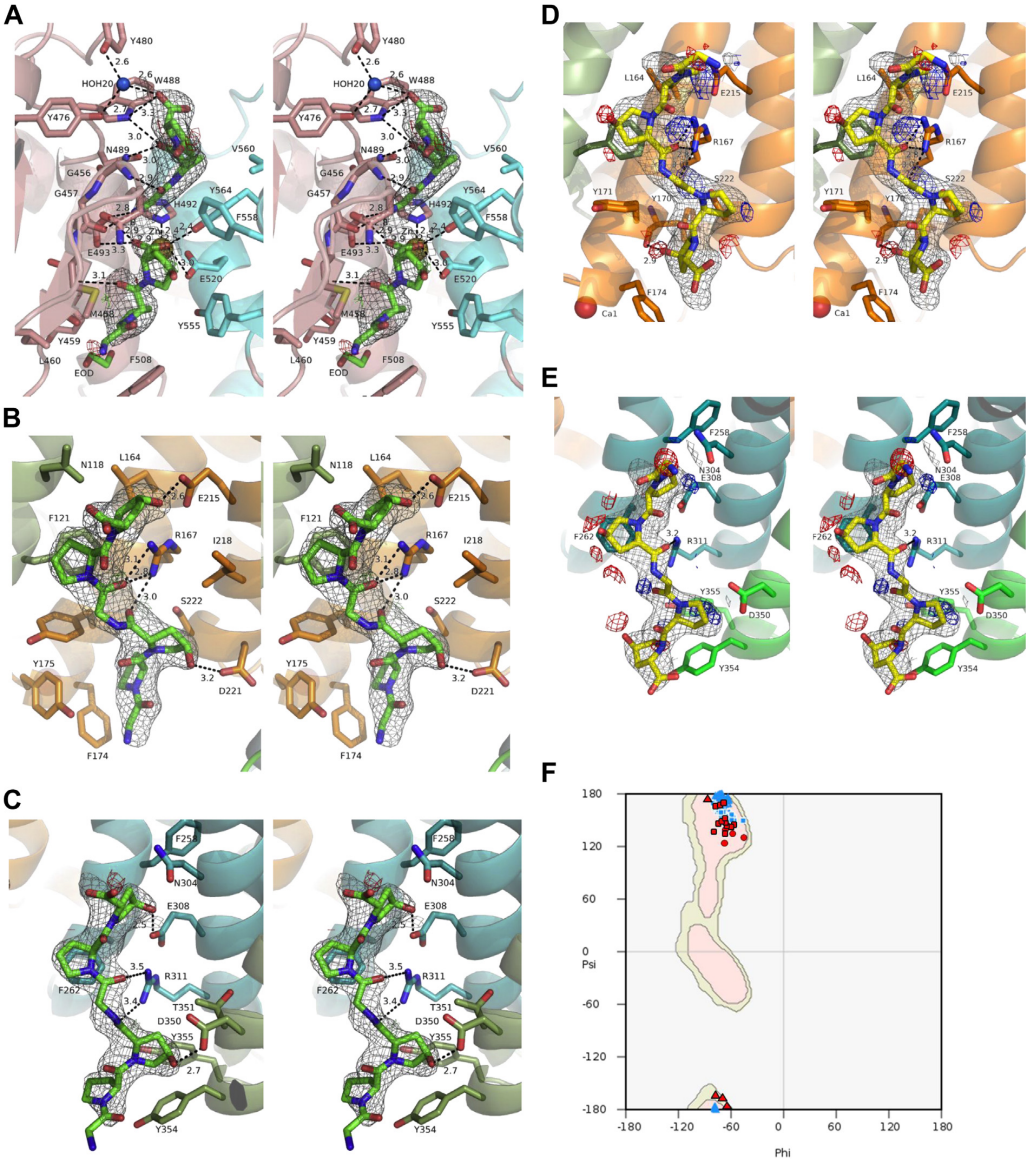
**Structure of Ghcol complexed with Gly-Pro-Hyp in *left* and *right* stereo drawings.***A*–*E*, close-up views of a, b, and c for Figure 1*C* are shown in (*A*–*C*), respectively. The 2*f*_o_–*f*_c_ omit map (*blue*) and *f*_o_–*f*_c_ map with model are shown at 1.0 s and ±2.8 s (*green* and *red*), respectively. The residues with hydrogen bonds and C–C contacts are labeled. No peaks of *f*_o_–*f*_c_ map appeared in (*A*–*C*). *D* and *E*, the bound six residues in the activator domain (a and b sites for Fig. 1*C*) are modeled in a reverse direction and refined. There are several *f*_o_–*f*_c_ map peaks. The omit map is shown in *gray color*. *F*, Ramachandran plot. The backbone torsional angles for the collagen triple helix model of (Pro-Pro-Gly)_10_ (PDB ID: 1K6F) are also plotted (*blue*). The angles for Gly, Pro, and Hyp are shown with *triangles*, *squares*, and *circles*, respectively. The favored (*thin red*) and the allowed (*thin green*) regions are for proline residues generated by COOT. Ghcol, collagenase from *Grimontia hollisae* strain 1706B; PDB, Protein Data Bank.

Figure 3, *A*–*C* shows the binding sites a, b, and c, respectively, in more detail with the omit 2*f*_o_–*f*_c_ map and the *f*_o_–*f*_c_ map with the peptide model. No peaks of the *f*_o_–*f*_c_ map appeared, indicating that these models are appropriate. In Figure 3*A*, the two peptides in the active site are clearly separated, suggesting that this structure might reflect the enzyme–product complex after cleavage of collagen. In Figure 3, *B* and *C*, the two peptides at the activator domain are partially overlapping; the O atom of Hyp in the first peptide and the N atom of Gly in the second peptide are located at almost the same position. This might be because these two atoms have multiple positions, and the average coordinates were obtained. There may be possibility that six-residue peptide contaminated or synthesized by reverse reaction during soaking preferentially bound to the binding site. We also modeled the six-bound residues of two Gly-Pro-Hyp in a reverse direction (Fig. 3, *D* and *E*). Several peaks appeared in the *f*_o_–*f*_c_ map, indicating that these models are inappropriate. Figure 3*F* shows the Ramachandran plot. The conformations of the Gly-Pro-Hyp peptides bound to Ghcol are almost the same as those of Pro-Pro-Gly repeats in a model collagen (Pro-Pro-Gly)_10_. We therefore propose that these three sites contribute to unwinding of the triple helix of collagen.

Crystallographic structures of the collagenase modules of clostridial collagenase isoforms, ColG, ColH, and ColT, have been previously reported to be strikingly similar (8, 9). In this study, the overall structures of Ghcol and ColG are shown to be similar with an RMSD of 1.56 to 2.38 Å (Table 2). However, Ghcol does not contain a domain corresponding to the helper subdomain in the peptidase domain of ColG. Comparison of the zinc-binding motifs of Ghcol and ColG (Fig. S5) revealed that both contain two His residues (His492 and His496 for Ghcol and His523 and His527 for ColG) and one Glu residue (Glu520 for Ghcol and Glu555 for ColG) that coordinate to the zinc ion, and the catalytic Glu residue and one water molecule are located near the zinc ion.

### Structures of calcium-binding sites of Ghcol

Ghcol has four calcium ions, one near the active site (Ca3 in Fig. 1*C*) and three distant from the active site (Ca1, Ca2, and Ca4 in Fig. 1*C*). In clostridial collagenase, one calcium ion was observed near the active site in ColH and ColT, whereas no calcium ions were observed in ColG (8, 9). Fig. S6, *A*−*D* shows the calcium-binding sites of Ghcol for Ca1 in the activator domain, for Ca2 in the upper-half subdomain in the peptidase domain, for Ca3 near the active site, and for Ca4 in the lower-half subdomain of the peptidase domain, respectively. Though no homology was observed among these four calcium-binding sites, they occur at the C-terminal end of α-helices except for Ca2. Fig. S6*E* shows the comparison of the Ca3-binding sites of Ghcol with the corresponding sites of ColH, ColT, and *Vibrio* collagenase VhaC. All these sites are similar, containing conserved one Glu, two Gly, and one Arg residues and two water molecules.

Clostridial collagenases consist of a collagenase module and a C-terminal segment. In ColH, the C-terminal segment comprises two polycystic kidney disease–like domains and a collagen-binding domain. Ohbayashi *et al.* (7) demonstrated that calcium ion plays an important role in the stability of full-length ColH expressed in *Esherichia coli*. They also conducted a small-angle X-ray scattering analysis, which revealed that the full-length ColH adopted a tapered shape with a swollen head and an elongated overall structure under calcium-chelated conditions (25). Ghcol consists of a collagenase module and a bacterial prepeptidase C-terminal segment. The role of calcium ions on Ghcol activity, stability, and structure will be investigated in future studies.

### Active-site structures of Ghcol

**Figure 4:**
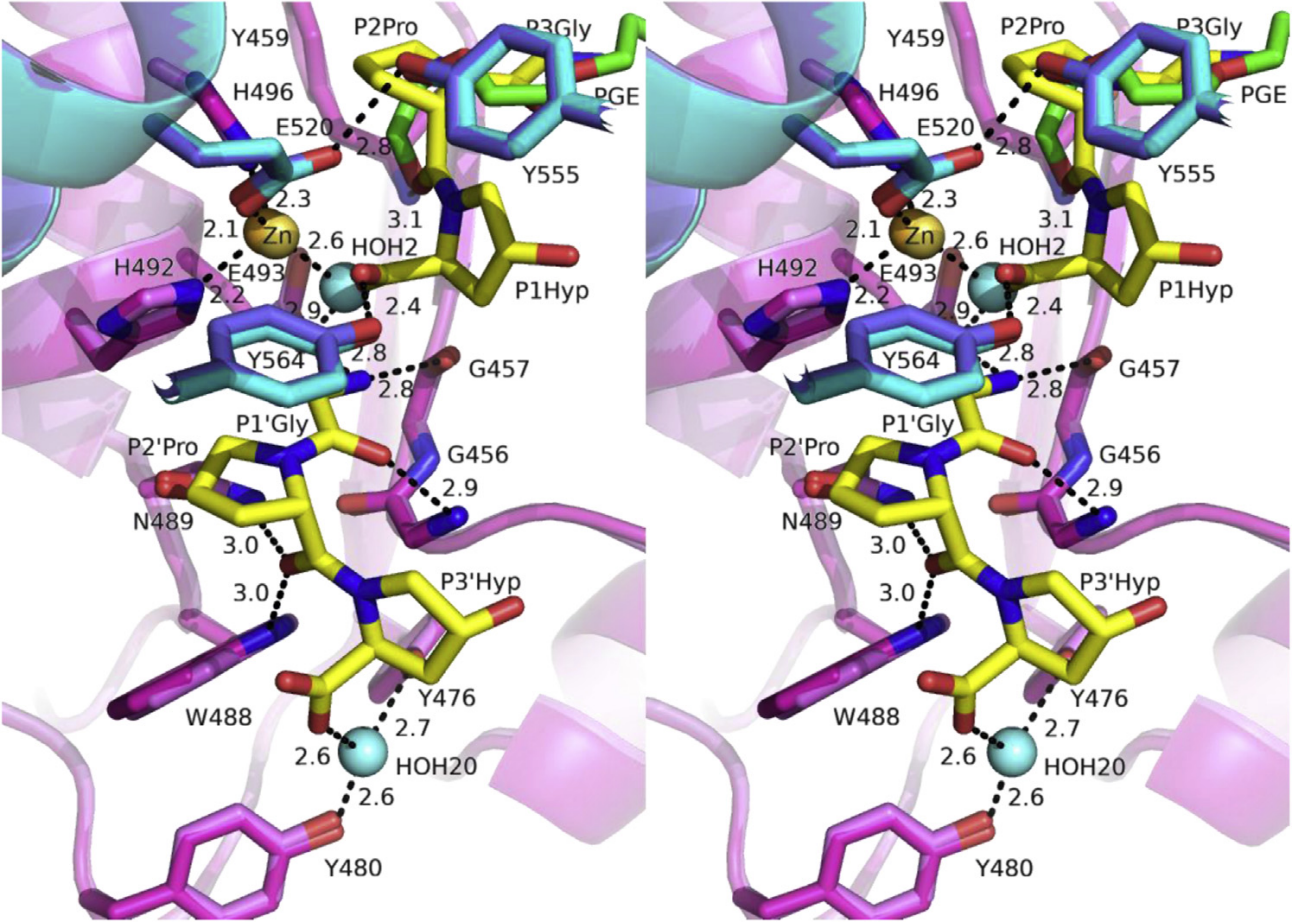
**Active-site structure of Ghcol (*left* and *right* stereo drawing).** Superposition of the active site of the ligand-free Ghcol (*pink* and *cyan* for the peptidase upper-half [C1] and lower-half [C2] subdomain, respectively) and Gly-Pro-Hyp-complexed Ghcol (*dark pink* and *dark cyan* for C1 and C2 subdomain, respectively). The catalytic zinc ion and the oxygen of water near the zinc ion are shown as a *yellow* (*orange* for ligand-free) and *cyan sphere*, respectively. The peptide is colored in *yellow*, and PGE (triethylene glycol) found in the ligand-free Ghcol is colored in *green*. Ghcol, collagenase from *Grimontia hollisae* strain 1706B.

Table 3 and Figure 3, *A*–*C* show the hydrogen bonds and C–C contacts between Ghcol and Gly-Pro-Hyp peptides. Notably, the Hyp side chains form more hydrogen bonds than those of Pro in all three binding sites. Figure 4 shows the active-site structure of Ghcol. His492, His496, and Glu520 coordinate to the zinc ion in the Ghcol active site. Zinc-bound water is present in the ligand-free Ghcol structure, whereas in the ligand-bound Ghcol, this water is replaced by the carboxyl oxygen of the Hyp at the P1 position.

**Table 3:** Hydrogen bonds and C–C contacts between Ghcol and Gly-Pro-Hyp

Gly-Pro-Hyp		Protein atom	Chain A		Chain B	
Chain	Atom		H-bond (Å)	Residues with C–C contact	H-bond (Å)	Residues with C–C contact
C/G	Gly1			Met458, Tyr459, Leu460, Phe508		Tyr459, Leu460, Phe508
	O Pro2	N Tyr459	3.1	Tyr459, His496, Phe508	3.0	Tyr459, His496, Phe508
	O Hyp3	OH Tyr564	2.4	Asn455, Glu520, Tyr555, Phe558	3.1	Asn455, Met458, Tyr555, Phe558
	O Hyp3	OE2 Glu520	3.0		3.8	
	OXT Hyp3	OE1 Glu493	2.9		3.5	
	OXT Hyp3	OE2 Glu493	3.2		3.2	
	OXT Hyp3	O Gly457	3.3		3.6	
	OXT Hyp3	Zn	2.8		2.6	
	O Hyp3	Zn	2.5		3.2	
D/H	N Gly1	O Gly457	2.8	Asn489, Gly456, Tyr564, His492	2.9	Asn489, Tyr564, Gly456, His492
		OE2 Glu493	2.9		2.7	
	O Gly1	N Gly456	3.0		2.9	
	O Pro2	NE1 Trp488	3.0	Tyr564, Gly456, Val560, Tyr564	3.0	Tyr564, Gly456, Tyr564, His492
		ND2 Asn489	3.0	Hi492, Glu524, Asn489	2.9	Asn489
	OXT Hyp3	NE1 Trp488	3.3	Gly456, Trp488, Phe558, Asn455	3.2	Gly456, Trp488, Phe558
	OXT Hyp3	HOH20	2.6	Val560		
	HOH20	OH Tyr476	2.6			
	HOH20	OH Tyr480	2.7			
E/I	Gly1			Phe174		Phe174
	Pro2			Tyr170, Ser222, Tyr171, Phe174		Tyr170, Tyr171, Phe174
	O Hyp3	NH1 Arg167	3.0	Tyr171, Ile218, Asp221, Ser222	3.2	Tyr171, Ile218, Ser222
	OD1 Hyp3	OD2 Asp221	3.2		2.9	
	O Gly4	NH1 Arg167	2.8	Phe121, Tyr171, Arg167	3.0	Phe121, Tyr171
	O Gly4	NH2 Arg167	3.1		3.1	
	Pro5			Phe121, Asn118		Phe121, Asn118
	OD1 Hyp6	OE2 Glu215	2.6	Leu164, Asn118	2.7	Leu164, Asn118
		NH2 Arg167	3.1		3.2	
F/J	Gly1			Tyr354		Tyr354
	Pro2			Tyr354		Tyr354
	O Hyp3	NH1 Arg311	3.4	Thr351, Asp350, Tyr354, Tyr355	2.8	Thr351, Asp350, Tyr354, Tyr355
	OD1 Hyp3	OD1 Asp350	2.7		3.2	
	O Gly4	NH2 Arg311	3.5	Phe262	3.1	Phe262
	Pro5			Phe262		Phe262
	OD1 Hyp6	OE2 Glu308	2.5	Phe258, Phe262, Asn304, Glu308	2.4	Phe258, Phe262, Asn304, Glu308
						Thr259

Chain A and B designate Ghcol of the Gly-Pro-Hyp-bound Ghcol. Chain C and G designate the one of the two Gly-Pro-Hyp molecules bound at the active site of Ghcol in chain A and B, respectively. Chain D and H designate the other one bound at the active site of Ghcol in chain A and B, respectively. Chain E and I designate two Gly-Pro-Hyp molecules bound at Act 1 of Ghcol in chain A and B, respectively. Chain F and J designate those at Act 2 of Ghcol in chain A and B, respectively.

Figure 4 also shows the Ghcol active-site residues thought to be important for catalysis. Three Tyr residues, Tyr476, Tyr555, and Tyr564 are located in the active site. The OH groups of Tyr555 and Tyr564 protrude to the zinc ion. The OH groups of Tyr476 and Tyr480 form water-mediated hydrogen bonds with OXT of Hyp at P3’. Tyr555 is involved in hydrogen bonding with OE1 atom of Glu520 both in the ligand-free and ligand-bound Ghcols (2.8 Å), and Tyr564 forms a hydrogen bond with O atom of Hyp in the ligand-bound Ghcol. Previous sequence comparison of Ghcol with ColH, ColG, and ColT suggested that the conserved Tyr568 residue may play an important role in catalysis in Ghcol (21). However, this is unlikely because Tyr568 is distant from the active site (not shown).

Based on crystallographic analysis of clostridial collagenase, it has been shown that the C1 and C2 subdomains, which constitute the active site, undergo contraction at different levels (ColG > ColT > ColH) upon the binding of a peptidic inhibitor (9). However, as shown in Figure 4, the binding of tripeptides does not significantly change the structure of the Ghcol active site. This may be explained by that crystal of Ghcol complexed with Gly-Pro-Hyp was prepared by soaking of Gly-Pro-Hyp into the crystal of ligand-free Ghcol, where conformation changes were restricted by crystal packing.

The crystal structure of *Vibrio* collagenase VhaC has been recently reported (24). Similar to Ghcol and clostridial collagenase, *Vibrio harveyi* collagenase (VhaC) contains an activator domain, linker, peptidase domain, and one zinc ion, exhibiting a saddle-shaped structure. The overall structures of Ghcol and VhaC are strikingly similar (Fig. S7*A*) with RMSD values of 0.35–1.38 Å (Table 2), which are smaller than those between Ghcol and ColG, ColH, or ColT (1.56–2.38 Å). The superposition of activator domain of Ghcol, ColG, and VhaC revealed that around 40^°^ rotation is required to fit the peptidase domain of ColG to that of Ghcol or VhaC (Fig. S7*A*). These results strongly suggest that Ghcol and VhaC may exhibit similar catalytic mechanisms. Fig. S7, *B* and *C* shows the rigid body rotation of the C1 and C2 subdomains in the peptidase domain. It shows that rotation angle between Ghcol and VhaC and that between Ghcol and ColG are similar (10.3^°^ and 13.3^°^, respectively), suggesting that the petidase domains are well conserved in Ghcol, ColG, and VhaC.

### Insight into the mechanism of collagenase to cleave collagen

Collagenase is thought to locally unwind the triple-helical structure of collagen before hydrolyzing the peptide bonds (26, 27). Based on the saddle-shaped structure of clostridial collagenase, Eckhard *et al.* (8) proposed a chew-and-digest mechanism for its catalytic action. In this mechanism, the triple helical collagen was suggested to be unwound first on interacting with the peptidase domain in the open conformational state of collagenase, followed by interaction with both the activator and peptidase domains in the closed state (8). On the other hand, Wang *et al.* (24) demonstrated that the binding of the collagen triple helix occurs only to the activator domain by isothermal titration calorimetry analysis. They concluded that the collagen triple helix first binds to the activator domain before unwinding and cleavage.

**Figure 5:**
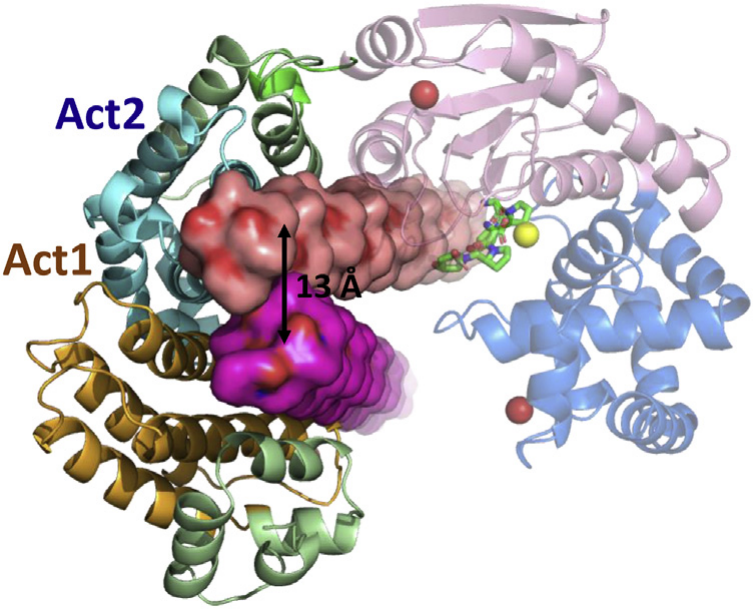
**Fitting of the collagen triple helix model (PDB:****1K6F****) to the bound peptides in Act1 and Act2.** The superposition was performed by the ligand fitting implemented in COOT. PDB, Protein Data Bank.

In the present study, Ghcol was observed to bind two Gly-Pro-Hyp molecules at each of three sites: the active site, Act1, and Act2. All the bound tripeptides exhibited the same conformation as the Pro-Pro-Gly units in collagen (Fig. 3*F*). The binding site of Act1 is 11 Å apart from Act2 and 23 Å apart from the active site. The distance between Act2 and the active site is 22 Å (Fig. 1*C*). Fitting the collagen triple helix (Protein Data Bank [PDB] ID: 1K6F) to the active-site peptide resulted in collision with the amino acid residues, suggesting that direct binding of the collagen triple helix to the active site of Ghcol is difficult. In contrast, the superposition of the collagen triple helix model (PDB ID: 1K6F) to the Gly-Pro-Hyp binding sites in Act1 and Act2 (Fig. 5) revealed the possible binding of the two collagen triple helices to the activator domains. The distance between the two collagen triple helices is 13 Å, which is close to the distance between the two collagen triple helices observed in the crystal structure of collagen (PDB ID: 1K6F). This suggests that the activator domain unwinds the collagen fibril to the triple helix state. The unwinding of the triple helix may occur *via* a conformational change mediated by the open–close motion of the domain as estimated by Eckhard *et al.* (8) and Wang *et al.* (24).

Unlike bacterial collagenases, the mechanism of mammalian collagenase to cleave collagen has been extensively studied. Collagenolytic matrix metalloproteinases (MMPs) consist of catalytic (CAT) and hemopexin-like (HPX) domains. Based on the structural analysis of the complex of MMP-1 and collagen-model triple-helical peptide (THP) consisting of three (GPO)_4_-GPQGIAGQRGVVGLO-(GPO)_4_ chains, the following cleavage mechanism has been proposed (28, 29): MMP-1 is in equilibrium between open and closed forms. The HPX domain of the open-form MMP-1 binds Val23-Leu26 in two of the three THP chains. Then, the CAT domain binds Gly16-Ile17 of one chain of the open form of MMP-1, which is subsequently cleaved. To explore the cleavage mechanism of Ghcol and other bacterial collagenases, structural analysis of THP–collagenase complex is required

### Activities of Ghcol and clostridial collagenases toward collagen, gelatin, and synthetic peptides

Commercially available ColH and ColG were used to compare the activity of clostridial collagenase with that of Ghcol. Both proteins migrated as a 120-kDa band on SDS-PAGE under reducing conditions (Fig. S3), suggesting that they contained the C-terminal polycystic kidney disease–like domain and collagen-binding domain, unlike Ghcol.

**Figure 6:**
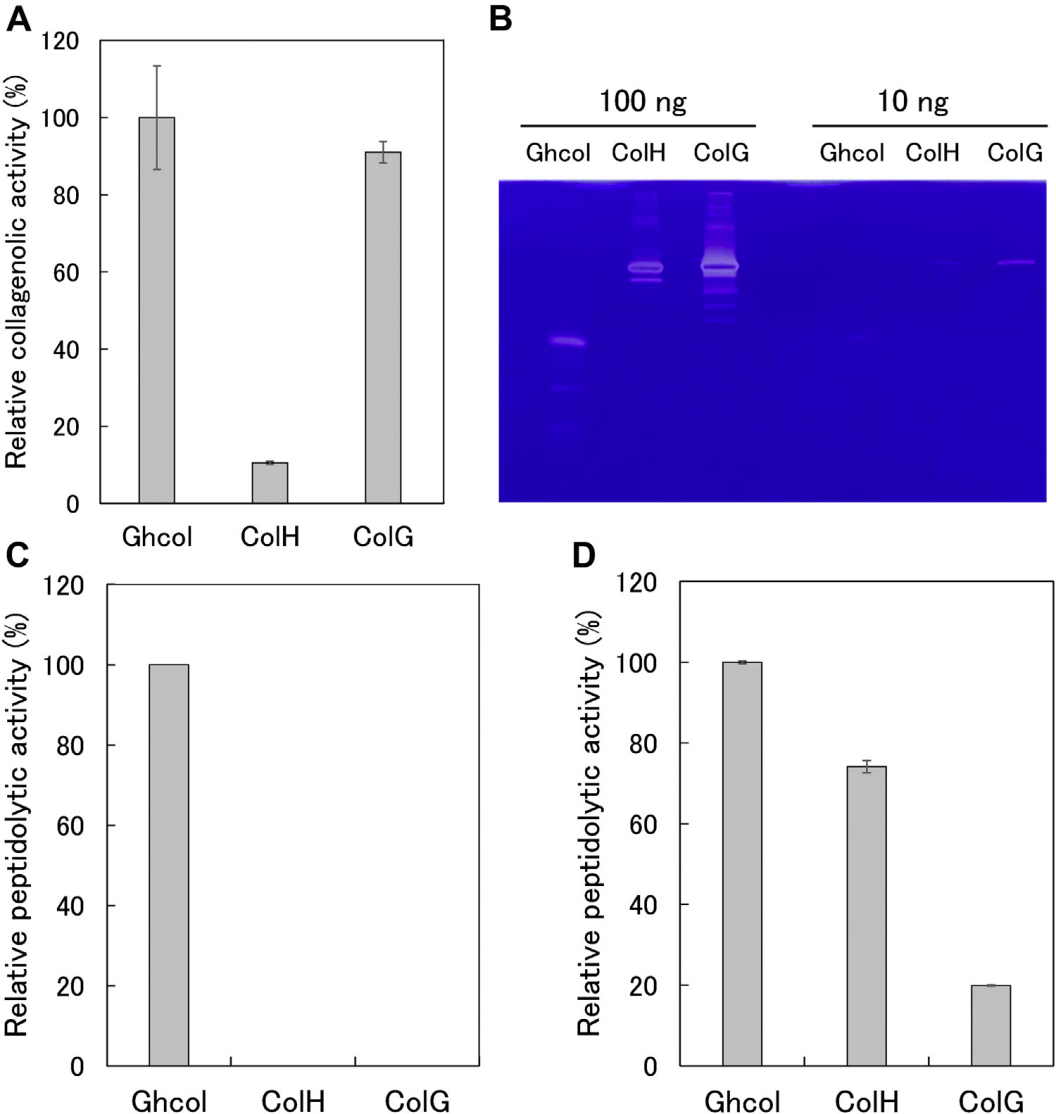
**Activities of Ghcol and clostridial collagenase.***A*, FITC-collagen hydrolytic activities. The reaction was carried out in 50 mM Tris–HCl buffer (pH 7.5), 5 mM CaCl_2_, 200 mM NaCl, and 1.25 mM acetic acid with 0.1 μg/ml Ghcol, 1.0 μg/ml ColH, or 0.1 μg/ml ColG in the presence of 0.025% FITC-labeled type I collagen at 35 °C. *B*, gelatin-hydrolyzing activities. A 100 ng or 10 ng of Ghcol, ColH, or ColG were applied to 12.5% polyacrylamide gel containing 0.063% gelatin. Coomassie brilliant blue–stained gels are shown. *C*, hydrolysis of MOCAc-KPLGL(Dpa)-AR peptide. The reaction was carried out in 5 mM Hepes–NaOH buffer (pH 7.0) with 1.0 μg/ml Ghcol, 100 μg/ml ColH, or 100 μg/ml ColG in the presence of 1.0 μM MOCAc-KPLGL(Dpa)-AR at 25 °C. *D*, hydrolysis of FALGPA. The reaction was carried out in 100 mM Hepes–NaOH buffer (pH 7.5), 200 mM NaCl, 10 mM CaCl_2_, 10 μM ZnCl_2_ with 0.50 μg/ml Ghcol, 1.0 μg/ml ColH, or 10 μg/ml ColG in the presence of 80 μM FALGPA at 25 °C. Relative activity is the activity compared with that of Ghcol. Error bars indicate SD values of triplicate determination. FALGPA, *N*-[3-(2-furyl)acryloyl]-Leu-Gly-Pro-Ala; Ghcol, collagenase from *Grimontia hollisae* strain 1706B; MOCAc-KPLGL(Dpa)-AR, (7-methoxycoumarin-4-yl)acetyl-Lys-Pro-Leu-Gly-Leu-[*N*^3^-(2,4-dinitrophenyl)-2,3-diaminopropionyl]-Ala-Arg.

First, we analyzed the ability of these three collagenases to hydrolyze FITC-collagen. As shown in Figure 6*A*, ColG exhibited a specific activity comparable to that of Ghcol, whereas the specific activity of ColH was only 10% that of Ghcol. Unlike with FITC-collagen hydrolysis, both ColH and ColG exhibited gelatin-hydrolyzing activity, and ColG appears more active than Ghcol (Fig. 6*B*). Next, we analyzed the hydrolytic activities toward the fluorogenic peptide substrate (7-Methoxycoumarin-4-yl)acetyl-Lys-Pro-Leu-Gly-Leu-[*N*^3^-(2,4-dinitrophenyl)-2,3-diaminopropionyl]-Ala-Arg (MOCAc-KPLGL(Dpa)-AR) (Fig. S9*A*). When the reaction started, the fluorescence intensity at 400 nm (*FI*_400_) of the reaction solution increased with increasing time (Fig. S10*A*). As shown in Figure 6*C*, the specific activities of ColH and ColG were less than 1% of that of Ghcol. As shown in Fig. S9*A*, MOCAc-KPLGL(Dpa)-AR has bulky residues at P3 and P3’. The result suggests that Ghcol has the large space for substrate binding in the active site. Finally, we analyzed the ability of the collagenases to hydrolyze *N*-[3-(2-furyl)acryloyl]-Leu-Gly-Pro-Ala-OH (FALGPA) (Fig. S9*B*). In this assay, the rate of decrease in absorbance at 322 nm of the reaction solution corresponded to *v*_o_ and was proportional to the enzyme concentration (Fig. S10*B*). As shown in Figure 6D, the specific activities of ColH and ColG were 70% and 20% of that of Ghcol, respectively. In FITC-collagen digestion, ColG exhibited higher activity than ColH, whereas ColG exhibited lower activity on FALGPA than ColH. This may be because the selective loop of ColH covers the active site more substantially than that of ColG (8, 9).

**Figure 7:**
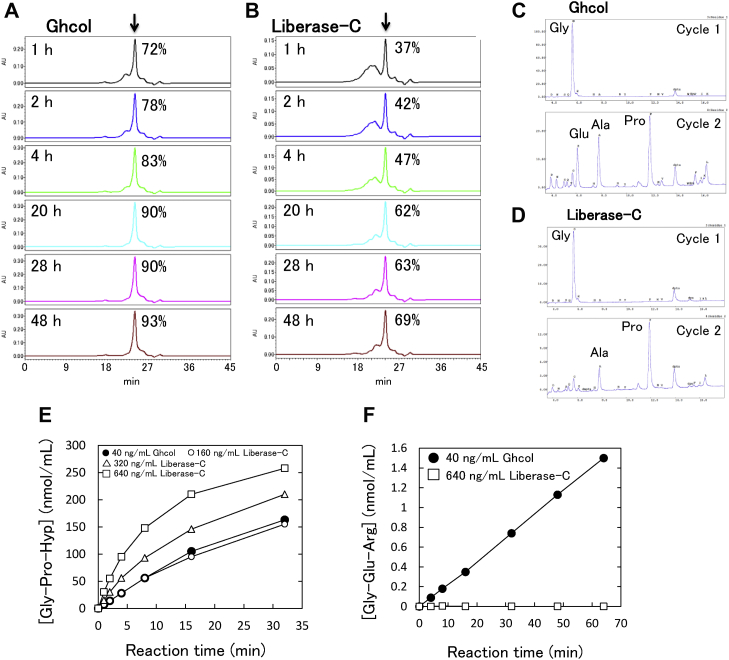
**Activities ofGhcol and clostridial collagenase Liberase-C.***A* and *B*, time course of the collagen-hydrolysis reaction. Elution patterns of gel filtration column chromatography of the reaction solution with Ghcol (*A*) and Liberase-C (*B*) are shown. The *arrow* indicates the position where tripeptides were eluted. Relative values of the area corresponding to tripeptides compared with total area are shown in percentage. *C* and *D*, analysis of tripeptides. The tripeptides in the reaction solution at 20 h in (*A*) and (*B*) were collected and subjected to Edman degradation (*C* and *D*, respectively). Elution pattern of reversed-phase HPLC of the Edman degradation products at cycle 1 and 2 is shown. *E* and *F*, hydrolysis of hexapeptides. The reaction was carried out in 50 mM Tris–HCl buffer (pH 7.5), 1 mM CaCl_2_ with Ghcol or Liberase-C in the presence of 500 nmol/ml Gly-Pro-Hyp-Gly-Pro-Hyp (*E*), or Gly-Glu-Arg-Gly-Phe-Hyp (*F*) at 37 °C for indicated durations (1–70 min). The concentrations of Gly-Pro-Hyp and Gly-Glu-Arg were determined by LC–MS/MS. Time courses of Gly-Pro-Hyp (*E*) and Gly-Glu-Arg (*F*) concentrations in the reaction solution are shown. Ghcol, collagenase from *Grimontia hollisae* strain 1706B.

We compared collagenolytic activities of Ghcol and clostridial collagenase, by hydrolyzing collagen using Ghcol or Liberase-C, which is a mixture of ColH and ColG and analyzing the reaction products using gel-filtration HPLC (Fig. 7, *A* and *B*). The reaction products at 1 h exhibited two peaks with retention times of 21 and 24 min. The former peak corresponded to peptides containing more than six amino acid residues, whereas the latter peak corresponded to tripeptides. With increasing reaction time (1–48 h), the former peak area decreased, and the latter peak area increased (72–93% for Ghcol and 37–69% for Liberase-C), indicating that collagen digestion continued. The former peak hardly appeared in the Ghcol reaction at 20 to 48 h, whereas, it clearly appeared in the Liberase-C reaction, suggesting that Ghcol degrades collagen more efficiently than Liberase-C. N-terminal amino acid sequence analysis of the reaction products at 20 h revealed that the peak corresponding to Glu appeared in the second cycle for Ghcol (Fig. 7*C*) but did not appear for Liberase-C (Fig. 7*D*). In X position of Gly-X-Y repeat in collagen, Pro is the most, Ala is the second most, and Glu is the third most abundant residues (30). Van Wart and Steinbrink (30) reported that clostridial collagenase has a preference for Pro and Ala, but not Glu, for P2 and P2′ sites. Eckhard *et al.* (31) reported that ColH, ColG, and ColT do not favor substrates that contain Asp, Glu, Lys, or Arg at P2 or P2′ sites. In contrast, our results suggest that Ghcol cleaves collagen even when Glu residues occupy the P2 and P2′ sites. When the degradation products by Liberase-C in the former peak were fractionated by gel filtration chromatography and further purified by reverse-phase chromatography, a nonapeptide including Glu, Gly-Gln-Arg-Gly-Glu-Arg-Gly-Phe-Hyp (bovine Col α(I) chain precursor 964–972) was identified. Therefore, we next hydrolyzed Gly-Pro-Hyp-Gly-Pro-Hyp (Fig. 7*E*) and Gly-Glu-Arg-Gly-Phe-Hyp (Fig. 7*F*). With increasing reaction time, the Gly-Pro-Hyp concentration increased for both Ghcol and Liberase-C (Fig. 7*E*), whereas the Gly-Glu-Arg concentration increased only for Ghcol (Fig. 7*F*). These results suggest that Ghcol, unlike clostridial collagenase, favors substrates that contain Glu at the X position of collagen, explaining that Ghcol degrades collagen more efficiently than clostridial collagenase.

**Figure 8:**
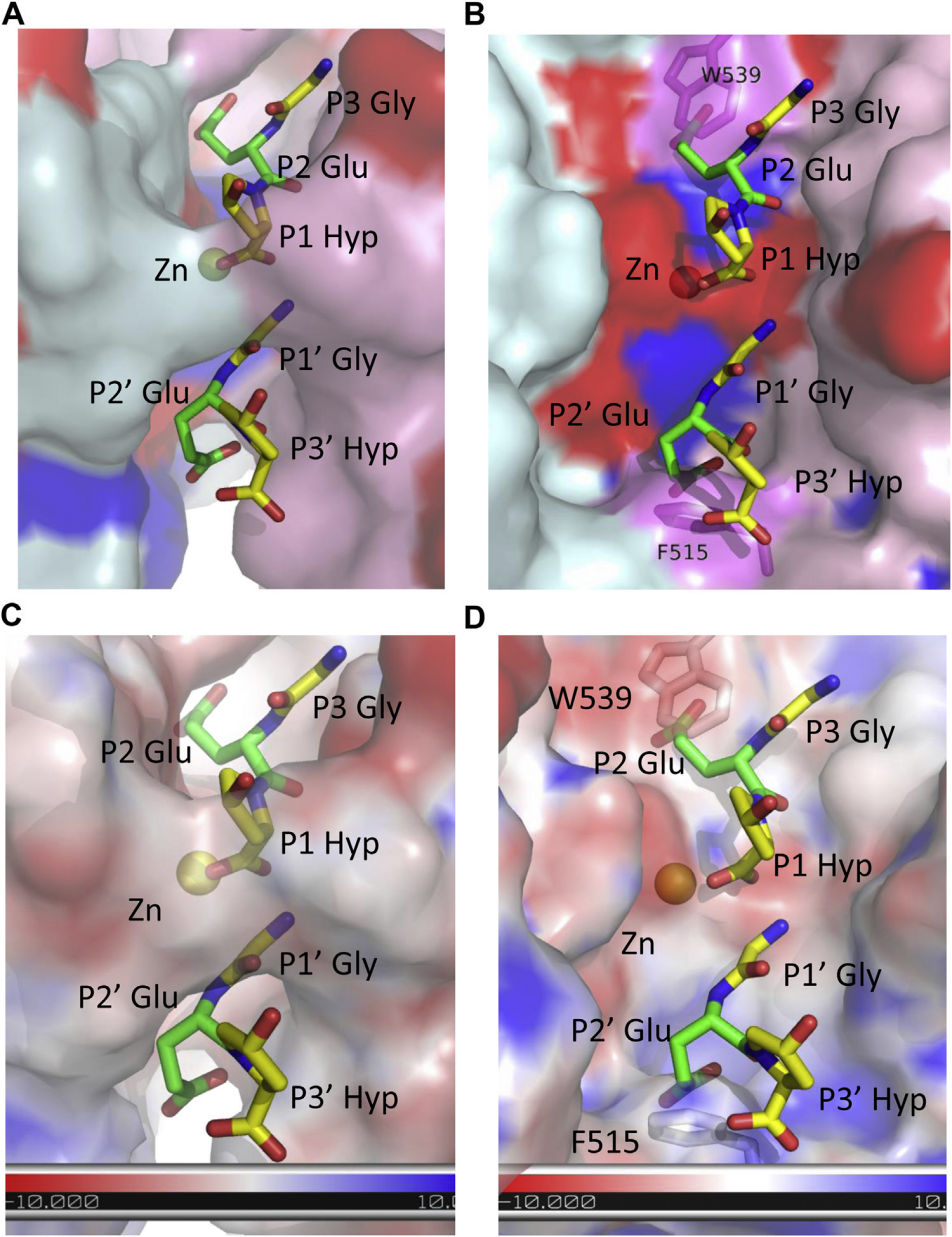
**Comparison of the surface of Ghcol and ColG.** The surface of Ghcol (*A* and *C*) and ColG (*B* and *D*) with Gly-Glu-Hyp peptides. The peptides are placed on the active site of ColG after fitting the peptidase domain of ColG to Ghcol. *A* and *B*, the surface is colored in *pink* and *cyan* for the peptidase upper-half (C1) and lower-half (C2) subdomain, respectively, except for acidic (*red*) and basic (*blue*) amino acids. *C* and *D*, the electrostatic surface potential density is colored gradient from *red* (−10 kT/e) to *blue* (10 kT/e). Ghcol, collagenase from *Grimontia hollisae* strain 1706B.

To explore the mechanism of the differences observed in Figures 6 and 7 between Ghcol and ColG, we compared the surfaces of Gly-Glu-Hyp-bound Ghcol and ColG (Fig. 8). The former structure was made by replacing Gly-Pro-Hyp with Gly-Glu-Hyp. The latter structure was made by placing Gly-Glu-Hyp on the active site of ColG after fitting the peptidase domain of ColG to Ghcol. Ghcol has enough space to accommodate the side chain of Glu at the P2 and P2′ positions, whereas there is a collision with the side chains of Trp539 and Phe515 in the case of ColG. In addition, compared with ColG, the active site of Ghcol is more hydrophobic. These findings suggest that Ghcol might exhibit broad specificity for P2 and P2′ residues in the substrate. This might explain that Ghcol degrades collagen more efficiently than clostrical collagenase.

### Activities of Ghcol variants toward collagen, gelatin, and synthetic peptides

Glu493 in the zinc-binding motif H^492^EYVH^496^ of Ghcol has been suggested to be the catalytic residue responsible for acidic p*K*_a_ (p*K*_e1_) (21, 23). In this study, Tyr476, Tyr555, and Tyr564, which are located in the active site, are suggested to be important for catalysis (Fig. 4). To examine the roles of these residues in more detail, we expressed Ala88−Gln767 of WT Ghcol and four variants, Y476A, Y555A, Y564A, and E493A, with an N-terminal Sec signal peptide using the *B. chosinensis* expression system (Fig. S2) and purified them from the culture supernatant. The purified preparations exhibited 62- and 74-kDa bands on SDS-PAGE under reducing conditions (Fig. S8).

**Figure 9:**
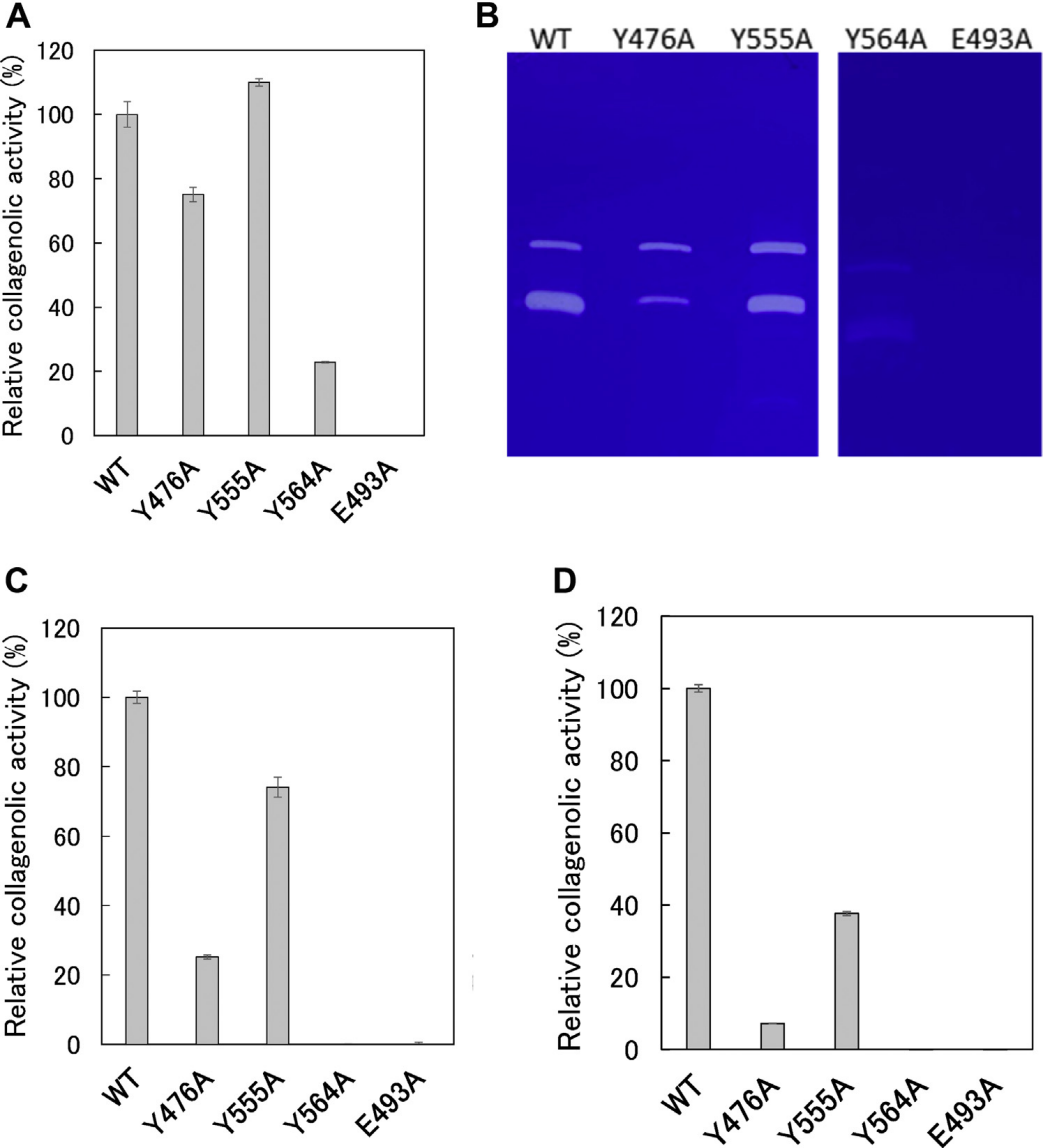
**Activities of Ghcol variants.***A*, FITC-collagen hydrolytic activities. The reaction was carried out in 50 mM Tris–HCl buffer (pH 7.5), 5 mM CaCl_2_, 200 mM NaCl, and 1.25 mM acetic acid with 0.50 μg/ml WT, 0.50 μg/ml Y476A, 0.50 μg/ml Y555A, 1.0 μg/ml Y564A, or 100 μg/ml E493A, in the presence of 0.025% FITC-labeled type I collagen at 35 °C. *B*, gelatin-hydrolyzing activities. About 10 μg/ml WT, 10 μg/ml Y476A, 10 μg/ml Y555A, 100 μg/ml Y564A, or 100 μg/ml E493A were applied to 12.5% polyacrylamide gel containing 0.063% gelatin. Coomassie brilliant blue–stained gels are shown. *C*, hydrolysis of MOCAc-KPLGL(Dpa)-AR peptide. The reaction was carried out in 5 mM HEPES–NaOH buffer (pH 7.0) with 1.0 μg/ml WT, 3.0 μg/ml Y476A, 1.0 μg/ml Y555A, 100 μg/ml Y564A, or 100 μg/ml E493A, in the presence of 1.0 μM MOCAc-KPLGL(Dpa)-AR at 25 °C. *D*, hydrolysis of FALGPA. The reaction was carried out in 100 mM Hepes–NaOH buffer (pH 7.5), 200 mM NaCl, 10 mM CaCl_2_ 10 μM ZnCl_2_ with 1.0 μg/ml WT, 10 μg/ml Y476A, 1.0 μg/ml Y555A, 57 μg/ml Y564A, or 10 μg/ml E493A, in the presence of 80 μM FALGPA at 25 °C. Error bars indicate SD values of triplicate determination. FALGPA, *N*-[3-(2-furyl)acryloyl]-Leu-Gly-Pro-Ala; MOCAc-KPLGL(Dpa)-AR, (7-Methoxycoumarin-4-yl)acetyl-Lys-Pro-Leu-Gly-Leu-[*N*^3^-(2,4-dinitrophenyl)-2,3-diaminopropionyl]-Ala-Arg; Ghcol, collagenase from *Grimontia hollisae* strain 1706B.

Figure 9, *A* and *B* shows the hydrolytic activities in the hydrolysis of FITC-collagen and gelatin, respectively. Y476A and Y555A had similar activity as WT, Y564A exhibited reduced activity, and E493A lacked this activity. Figure 9, *C* and *D* shows the activity on MOCAc-KPLGL(Dpa)-AR and FALGPA, respectively. Y555A and Y476A showed reduced activity compared with WT, whereas Y564A and E493A lacked this activity. In other words, mutation of Tyr564 affected catalysis rather than substrate recognition, whereas mutation of Tyr476 or Tyr555 affected substrate recognition rather than catalysis. This might be explained by that Tyr564 is closer to the zinc ion than Tyr476 and Tyr555, whereas Tyr476 and Tyr555 are closer to Gly-Pro-Hyp than Tyr564 (Fig. 4). These results suggest that Glu493 is indispensable for catalysis and that Tyr476, Tyr555, and Tyr564 contribute to catalysis and substrate recognition to varying degrees.

## Conclusion

The crystal structures of Ghcol revealed the following features. First, the activator and peptidase domains exhibit a saddle-shaped structure with one zinc ion and four calcium ions, which is similar to clostridial collagenase and strikingly similar to *Vibrio* collagenase VhaC. Second, Ghcol binds two peptides at each of the three sites: the active site and the two similar sites (Act1 and Act2) in the activator domain. Third, the activator domain contains two repeated subdomains to which collagen triple helix can bind. Finally, Ghcol has the large space for substrate binding at the S2 and S2′ sites. This explains its broad specificity for P2 and P2′ residues in the substrate. These findings are important for elucidating the mechanism of triple-helical collagen digestion by bacterial collagenases. The results from this study also explain the high catalytic activity and substrate specificity of Ghcol, encouraging its application in industry.

## Experimental procedures

### Protein expression and purification

Nucleic acid and protein sequences of Ghcol (Fig. S1) were obtained from the DNA Data Bank of Japan database (AB600550). The WT Ghcol (Ala88–Thr646) was expressed in *B. chosinensis* cells transformed with the expression plasmid (Fig. S2*A*) and purified from the supernatants as described previously (19). The expression plasmid for the WT Ghcol (Ala88−Gln767) (Fig. S2*B*) was previously described (23), from which the expression plasmids for Ghcol variants were constructed by QuikChange method using the primers listed in Table S1. The WT Ghcol and variants were expressed in *B. chosinensis* cells and purified from the supernatants as described previously (23).

### Crystallization and structural determination

One microliter of enzyme solution was mixed with 1 μl of reservoir solution (Crystal Screen I, II, and PEG/Ion Screen [Hampton Research]; Wizard Screen I and II [Emerald BioSystems]; and Structure Screen I and II [Molecular Dimensions]) and was equilibrated against 100 μl of reservoir solution at four or 20 °C, using the sitting drop vapor-diffusion method in a 96-well plate (Intelli-Plate; Art Robbins Instrument). Crystals were obtained after a few weeks in the following conditions: (i) ligand-free Ghcol: 20 °C, 9 mg/ml Ghcol, 75 mM Hepes, 1.2 mM CaCl_2_ at pH 7.5 for enzyme solution, and 0.1 M MES, 0.2 M Ca(CH_3_COO)_2_, 20% w/v PEG 8000 at pH 6.0 for reservoir solution and (ii) Ghcol complexed with Gly-Pro-Hyp: 4 °C, 10 mg/ml Ghcol, 75 mM Hepes, 1.2 mM CaCl_2_ at pH 7.5 for enzyme solution, and 0.1 M cacodylic acid, 0.2 M Ca(CH_3_COO)_2_, 18% PEG 8000 at pH 6.5 for reservoir solution, followed by the soaking of the crystal in the reservoir solution with 87 mM Gly-Pro-Hyp (Peptide Institute) for 30 min. Crystals were flash cooled in nitrogen gas stream at 100 K. Diffraction data were collected using synchrotron radiation on beamlines BL26B1 at SPring-8, after checked by an in-house detector system using Bruker Photon III detector coupled with Cu Kα radiation generated by IμS 3.0 generator (Bruker AXS). The collected diffraction data were processed with XDS (Max Planck Institute for Medical Research) (32). Molecular replacement was conducted using the predicted structure of Ghcol by AlphaFold2 (33, 34) as a search model. Structure refinement was conducted using COOT (35) and PHENIX (36). The electrostatic potential was calculated by PDB2PQR server (https://server.poissonboltzmann.org/pdb2pqr) and APBS (37) implemented in PyMOL (DeLano Sicentific).

### Enzyme assay

ColH and ColG were purchased from Meiji Seika Pharma. The concentrations of ColH and ColG were determined using Protein Assay CBB Solution (Nacalai Tesque, Inc) with bovine serum albumin (Nacalai Tesque, Inc) as a standard. *Clostridium histolyticum* collagenase, Liberase-C, was purchased from Roche Diagnostics. The concentration of Liberase-C was determined by the denoted weight. The *k*_cat_ value of Ghcol for type I collagen is 1.3 times higher than that of Liberase-C (22). Based on the *k*_cat_ value and molecular weights of both enzymes, enzyme/substrate ratios of 1% and 2.5% were used for Ghcol and Liberase-C, respectively.

Collagen and FITC-labeled type I collagen (FITC-collagen) was prepared as described previously (19). Collagen hydrolysis assay was carried out in accordance with a modified version of previously described method (20). Briefly, collagenases (6.0 μg/ml for Ghcol or 15.0 μg/ml for Liberase-C) were mixed with 50 mM Tris–HCl (pH 7.5) containing 0.6 mg/ml bovine type I collagen, 200 mM NaCl, and 5 mM CaCl_2_, and incubated at 30 °C for the time intervals shown in Figure 7, *E* and *F*. After heat shock to stop the enzymatic reaction, the collagenase digests were separated using gel filtration. Gel filtration analysis of the reaction products was carried out as follows: column, Superdex Peptide 10/30 HR (GE Healthcare); solvent, 0.1 M ammonium bicarbonate, 20% v/v acetonitrile; detector, and absorbance at 220 nm. FITC-collagen hydrolysis assay was carried out as described previously (19, 21, 23).

MOCAc-KPLGL(Dpa)-AR-NH_2_ (molecular mass of 1221.3 Da) (Fig. S9*A*) was purchased from Peptide Institute, and the hydrolysis assay was carried out as described previously (19, 21, 23). FALGPA (molecular mass of 476.53 Da) (Fig. S9*B*) was purchased from Bachem, and the hydrolysis assay was carried out as described previously (21). Gelatin was purchased from Sigma, and gelatin zymography was carried out as described previously (23).

Gly-Pro-Hyp-Gly-Pro-Hyp and Gly-Glu-Arg-Gly-Phe-Hyp were purchased from Peptide Institute. The hydrolysis assay was carried out in 50 mM Tris–HCl buffer (pH 7.5), 1 mM CaCl_2_ in the presence of 500 nmol/ml Gly-Pro-Hyp-Gly-Pro-Hyp or Gly-Glu-Arg-Gly-Phe-Hyp at 37 °C for 1 to 70 min, and stopped by adding formic acid to adjust 1% v/v. LC–MS/MS analysis of the reaction products was carried out using 3200QTRAP (Sciex) and Ascentis Express F5 (250 mm × 4.6 mm i.d.) (Agilent Technologies) as reported previously (38).

### Amino acid sequencing

N-terminal amino acid sequence analysis was performed as described previously (39). Briefly, collagenase digests after 20 h incubation were separated by gel filtration under aforementioned conditions, and tripeptide-containing fractions were collected. N-terminal sequence of the collected fractions was analyzed by a Procise 494 protein sequencer (Applied Biosystems) in pulsed-liquid mode.

## Data availability

The atomic coordinates and structure factors reported in this study were deposited in the PDB under accession code 7WSS for ligand-free Ghcol and 7XEB for Gly-Pro-Hyp-bound Ghcol.

## Supporting information

This article contains supporting information.

## Conflict of interest

The authors declare that they have no conflicts of interest with the contents of this article.
