# Supplementary material for: Crystal structure of Grimontia hollisae collagenase provides insights into its novel substrate specificity toward collagen
Source: J Biol Chem. 2022 Jun 6;298(8):102109. doi: 10.1016/j.jbc.2022.102109 (PMC9304777; doi:10.1016/j.jbc.2022.102109)
Supplement: Supplemental Table S1 [file mmc1.docx]

Table S1. Oligonucleotide.

Primers Sequences (5’-3’)

Y476A TCCCAATTTCATCGCGGCTGAAGCCAGCTATGCCA

Y476A_CP TGGCATAGCTGGCTTCAGCCGCGATGAAATTGGGA

Y555A GGTGTTTGACACGACGGCTGACGGTTTTGATGTGG

Y555A_CP: CCACATCAAAACCGTCAGCCGTCGTGTCAAACACC

Y564A TGATGTGGATCGCATCGCCCGATGGGGATATCTGG

Y564A_CP CCAGATATCCCCATCGGGCGATGCGATCCACATCA

E493A TCTGGAATCTTGAGCACGCTTACGTCCACTATTTG

E493A_CP CAAATAGTGGACGTAAGCGTGCTCAAGATTCCAGA
