# Supplementary material for: Crystal structure of Grimontia hollisae collagenase provides insights into its novel substrate specificity toward collagen
Source: J Biol Chem. 2022 Jun 6;298(8):102109. doi: 10.1016/j.jbc.2022.102109 (PMC9304777; doi:10.1016/j.jbc.2022.102109)
Supplement: Supplemental Figures S1–S10 [file mmc2.pdf]

1 ATGGAACTTAAAGCACTTGCGTTAACGGTATCAGCCCTGCTTGTGGGTCAGTCGGTGTATGCATCTGAGGTGTTTGCTCACCCGGGCATGCCGGTTCAA
1 M E L K A L A L T V S A L L V G Q S V Y A S E V F A H P G M P V Q
100 CAAAAGTTGCAGCCAAATTTGCTGCAGCAAAGTACCAGGCTTCAGCCAGAGCAACATATCCACGGGCTTGAACGCACGGATCGGCAATATCGTCCGACA
34 Q K L Q P N L L Q Q S T R L Q P E Q H I H G L E R T D R Q Y R P T
199 GATGCTACTCAGCAGCCGGAACCGCCAACGTTACTGAAACGTCAGGTATCGGTTCAGCAGGATGCGGTTGAACAGTGTGATCTCAGCCAGTTTCAGACG
67 D A T Q Q P E P P T L L K R Q V S V Q Q D A V E Q C D L S Q F Q T
298 ACCAGCAGTAACCAGTTGATGGCCGCGATTCGCCAGCAGGGCGCCAGTTGTGTCAATGCCTTGTTTAGTGCTGACACGGGAGTACAGGAAGCCGCGTTT
100 T S S N Q L M A A I R Q Q G A S C V N A L F S A D T G V Q E A A F
397 TCGTCCAACCACATGTATAACGTCGCACAATATACCCGAACACTGGCGCAGCAATATGCGGGCGGGGGCAGTGATGAACTGGAAGCCTTATATCTGTAT
133 S S N H M Y N V A Q Y T R T L A Q Q Y A G G G S D E L E A L Y L Y
496 CTTCGGGCGGGGTATTACGCTGAGTTTTATAACAGCAATATCACGTTTCTGTCGTGGGTGACGCCGGCAGTGAAAGGTGCCGTGGATGCGTTTGTCCAG
166 L R A G Y Y A E F Y N S N I T F L S W V T P A V K G A V D A F V Q
595 AATGCCCATTTTTATGACAACGGCGACGCTCATGGCAAGGTGCTGAACGAGGTGATCATCACGATGGACAGTGCTGGGCTACAGCATGCCTATCTCGAT
199 N A H F Y D N G D A H G K V L N E V I I T M D S A G L Q H A Y L D
694 GTAGTGACGCAGTGGCTGACCCGTTGGAATGCGCAATATGCCGAGCATTGGTATATGCGAAACGCAGTCAATGGTGTCTTTACTCTTTTGTTTGGCGGG
232 V V T Q W L T R W N A Q Y A E H W Y M R N A V N G V F T L L F G G
793 CAGTGGAACAATCAGTACACCAGCCTGATTGGCGAACAGACTGCGTTGGTGACGGCCTTACAGGCATTTGCGCTGGACCGTACGAAAGTGAACTCGCCA
265 Q W N N Q Y T S L I G E Q T A L V T A L Q A F A L D R T K V N S P
892 ACGGAGTTTATGGCGGCCAATGCGGCCAGAGAACTGGGGCGGTTAGCTCGCTACACGGACGCGACCATTGCGCCTAAAGTCACGGAAGGATTAACCGCG
298 T E F M A A N A A R E L G R L A R Y T D A T I A P K V T E G L T A
991 ATCTTCGGCCAGTATCCGTCCTATGGCGATGGAGATGCTATCTGGCTGGGGGCGGCGGATACGGCCTCTTATTATGCTGATTGCAGCCAGTTCAACATC
331 I F G Q Y P S Y G D G D A I W L G A A D T A S Y Y A D C S Q F N I
1090 TGTGGCTTTGAAGACGCGCTGCGTGATGCGGCGCTGAACCAGACTTTTATCTGTAGTGATACGATTAAAATTCGCTCACAGGATATGTCGCAGGCACAG
364 C G F E D A L R D A A L N Q T F I C S D T I K I R S Q D M S Q A Q
1189 CATCTCGCGGCTTGCGACAAAATGGCTTATGAAGAGTCATTTTTCCACACCACGCTTGAAACCGGTAATCAGCCGGTGGCTGATGATCATAATACGCAG
397 H L A A C D K M A Y E E S F F H T T L E T G N Q P V A D D H N T Q
1288 CTGCAGGTGAATATTTTTAATTCCGATACCGATTACGGTAAATATGCCGGTCCGATATTTGGGATTGATACCAACAACGGCGGTATGTACCTCGAGGGG
430 L Q V N I F N S D T D Y G K Y A G P I F G I D T N N G G M Y L E G
1387 AATCCGGCCAATGTGGGCAATATTCCCAATTTCATCGCGTATGAAGCCAGCTATGCCAACCCGGACCATTTTGTCTGGAATCTTGAGCACGAGTACGTC
463 N P A N V G N I P N F I A Y E A S Y A N P D H F V W N L E H E Y V
1486 CACTATTTGGATGGGCGGTTCAATATGTATGGCGATTTTGGTACGCCTACCGAGCTTGTGGTCTGGTGGAGCGAAGGGGTGGCCGAGTATGTGTCGCGG
496 H Y L D G R F N M Y G D F G T P T E L V V W W S E G V A E Y V S R
1565 GTAAATGATAATCCTCAGGCGATTGCCACCATCCAGGATGGTAGCACGTACACTCTGGCGCAGGTGTTTGACACGACGTATGACGGTTTTGATGTGGAT
529 V N D N P Q A I A T I Q D G S T Y T L A Q V F D T T Y D G F D V D
1684 CGCATCTACCGATGGGGATATCTGGCGGTGCGATTCATGTTTGAACGTCATCCTGATGAAGTTCAACGTATGCTGAGTGCCACCCGACAGGGACGCTGG
562 R I Y R W G Y L A V R F M F E R H P D E V Q R M L S A T R Q G R W
1783 GCAGAATACAAGGCGATCATTAGCGGTTGGGCAAATCAGTATCAGTCAGAATTTGCCCAATGGACCGAGGCGCTGGCGAAGGGCGACAGTGGCGCTGGG
595 A E Y K A I I S G W A N Q Y Q S E F A Q W T E A L A K G D S G A G
1882 AACGGTGAGGGGACAGGCTCCGGTAATGAAGGGGGCGGTGAATCTGGTGGTAATACCGGCTTGCCGGAAAACTGCGCAGTACTGCCAAAAATCAGTGAT
628 N G E G T G S G N E G G G E S G G N T G L P E N C A V L P K I S D
1981 GGGCGTTTAGCACTGGATGAAGCGGCCTGTCTGGCCGACACGGCTTCAGCGTCTGACGTATTGTGGTTCAGTATTCCGGCTGTCAGTGAATATCAGACC
661 G R L A L D E A A C L A D T A S A S D V L W F S I P A V S E Y Q T
2080 ATTGCCATTACGGCGGGCAACGGGACTGGCGACCTGACGCTGGAATACAGTAACCTGAATTGGCCAGATGGTACCAATGTGCAGGCATCATCGGCAAAT
694 I A I T A G N G T G D L T L E Y S N L N W P D G T N V Q A S S A N
2179 ATGGGTAACAGTGAATGCATTATTCTGGAACATCAGGCGAATTATTGGGGATATCTGAAAGTCTCGGGTTCCTTTGAAAATGCAGCGTTACTGGTGGAG
727 M G N S E C I I L E H Q A N Y W G Y L K V S G S F E N A A L L V E
2278 GCTGGCAGTAACCAGTGTCGTCAGTAA
760 A G S N Q C R Q *

Fig. S1. Nucleotide and amino sequences of Ghcol. The sequences are from GenBank accession number: HZ432578.1.

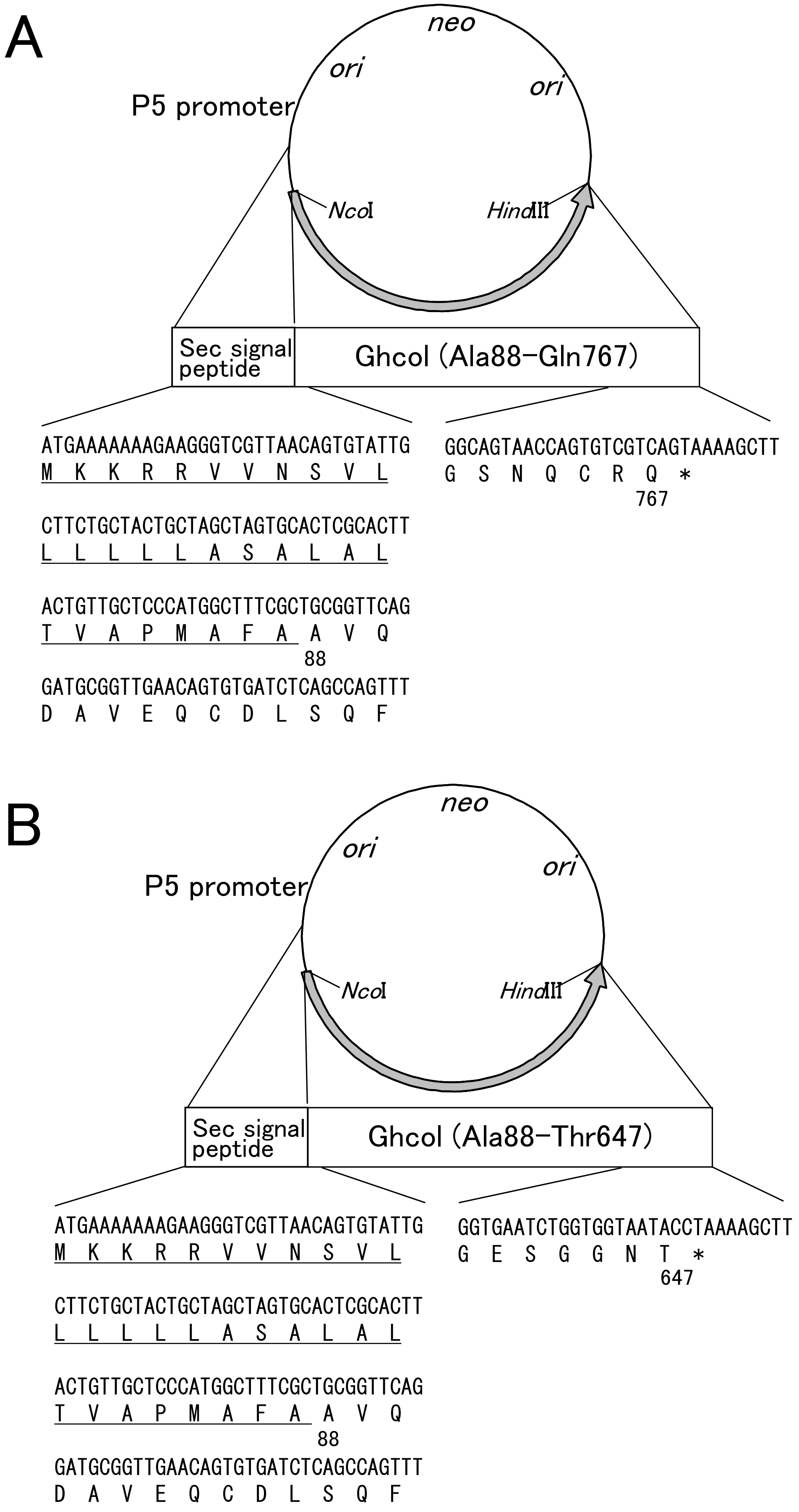

Fig. S2. Structures of expression plasmid. Sec signal peptide is underlined. (A) Ghcol (Ala88-Gln767). (B) Ghcol (Ala88-Thr647).

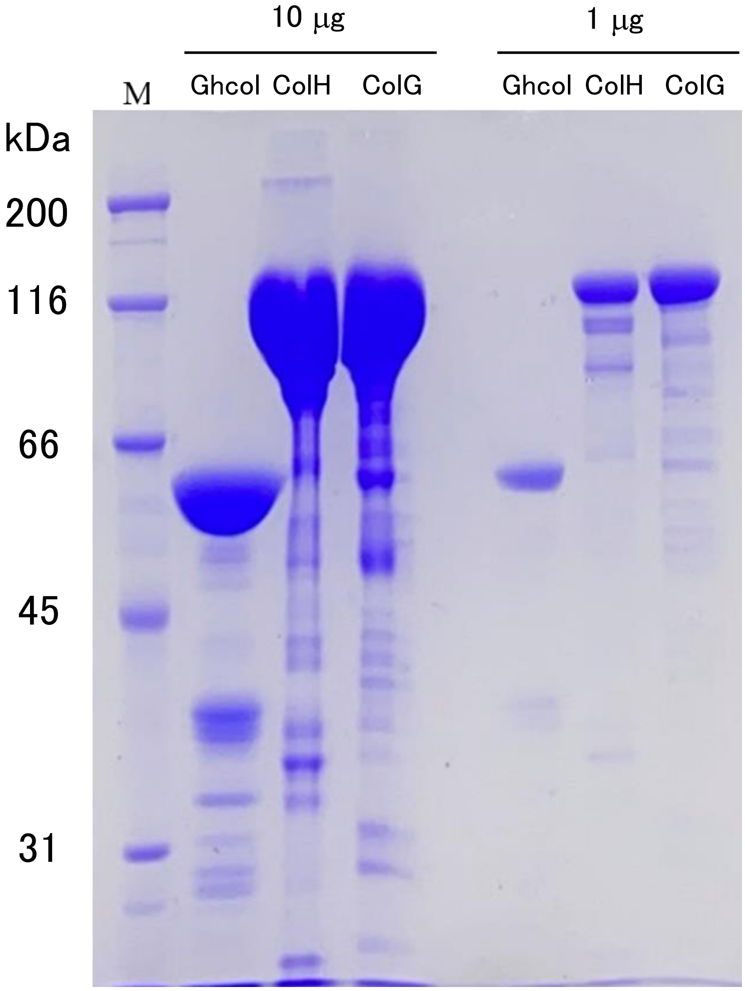

Fig. S3. SDS-PAGE of Ghcol, ColH, and ColG. Ten or 1 μg of protein was applied to 12.5% acrylamide gel.

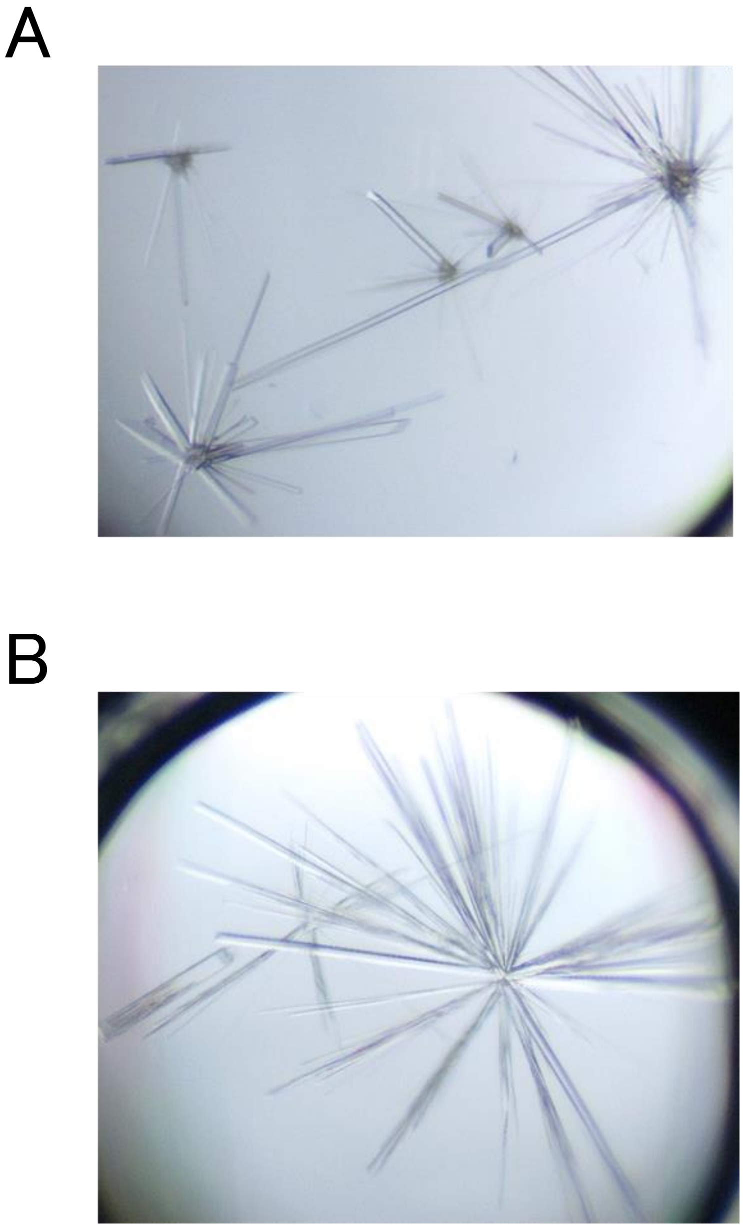

Fig. S4. Crystals of Ghcol. (A) Ligand-free Ghcol. (B) Ghcol complexed with Gly-Pro-Hyp.

A

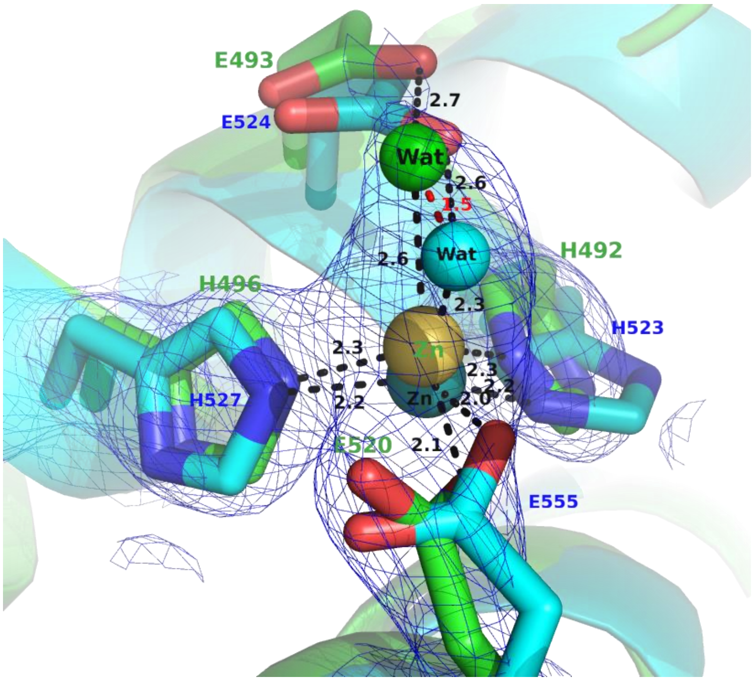

Fig. S5. Active-site structures of Ghcol (green) and ColG (cyan). The zinc ion and the oxygen of water of Ghcol are shown as a yellow and green sphere, respectively, and those of ColG are shown as cyan spheres. Amino acid residues that coordinate to the zinc ion and a putative catalytic Glu493 residue with the polder fo-fc omit map (Zn, Wat, H492, H496, and E520) contoured at 4.0 σ are shown.

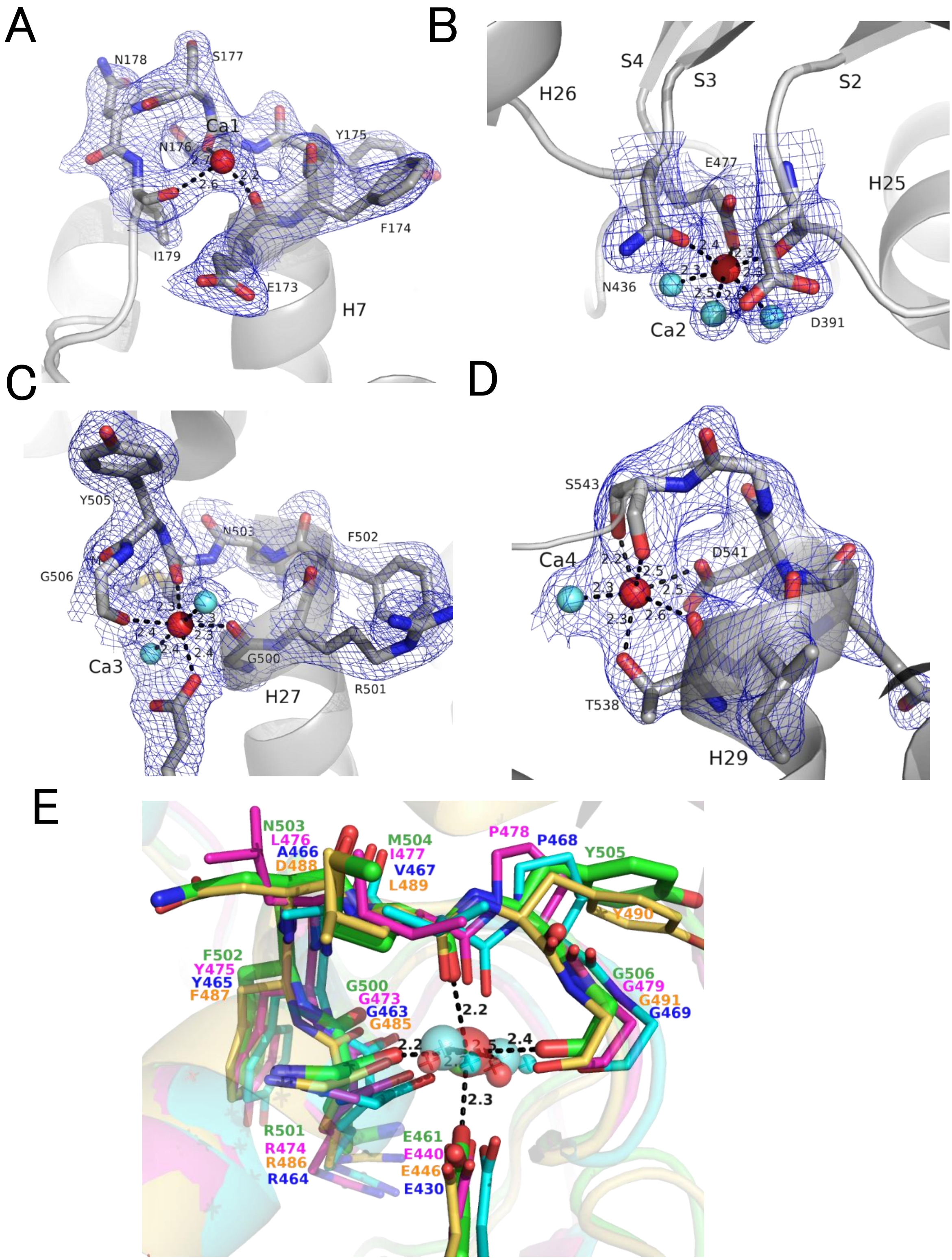

Fig. S6. (A–D) Calcium binding sites of Ghcol. (A) Ca1. (B) Ca2. (C) Ca3. (D) Ca4. The calcium ion is shown as a red sphere, and the oxygen of water is shown as a cyan sphere. Amino acid residues that coordinate to the calcium ion are shown. The 2fo–fc density map contoured at 1σ is shown. (E) Comparison of the Ca3-binding sites of Ghcol (green) with the corresponding sites of ColH (cyan), ColT (magenta), and VhaC (yellow).

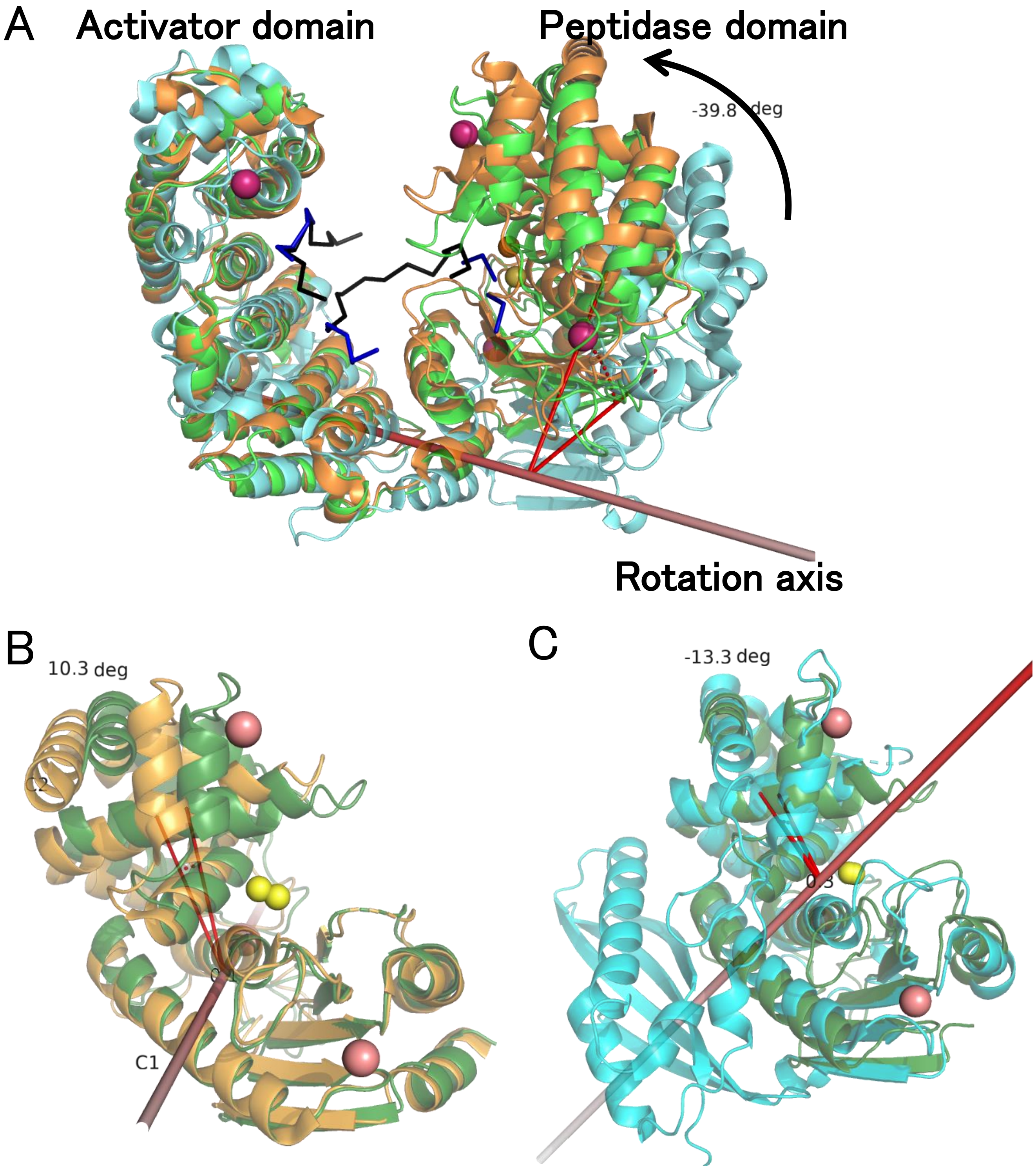

Fig. S7. Structural comparison of Ghcol with VhaC and ColG. Structures of Ghcol, VhaC (PDB ID 7ESI), and ColG (PDB ID 2Y50, without C-terminal extra domain) are shown in green, orange, and cyan, respectively. The zinc and calcium ions are shown as a yellow and red sphere, respectively. (A) Overall structures of Ghcol, VhaC, and ColG. The interacted peptides with VhaC, EPSQQVTEIYQHHA and DYAPTKLLPQQP, are shown in black, and that of Ghcol, Gly-Pro-Hyp, is in blue. (B) Peptidase domains of Ghcol and VhaC. (C) Peptidase domains of Ghcol and ColG. The red axis shows the rotation axis during the superposition of the C2 peptidase subdomain of ColG to that of Ghcol after fitting the C1 peptidase subdomains.

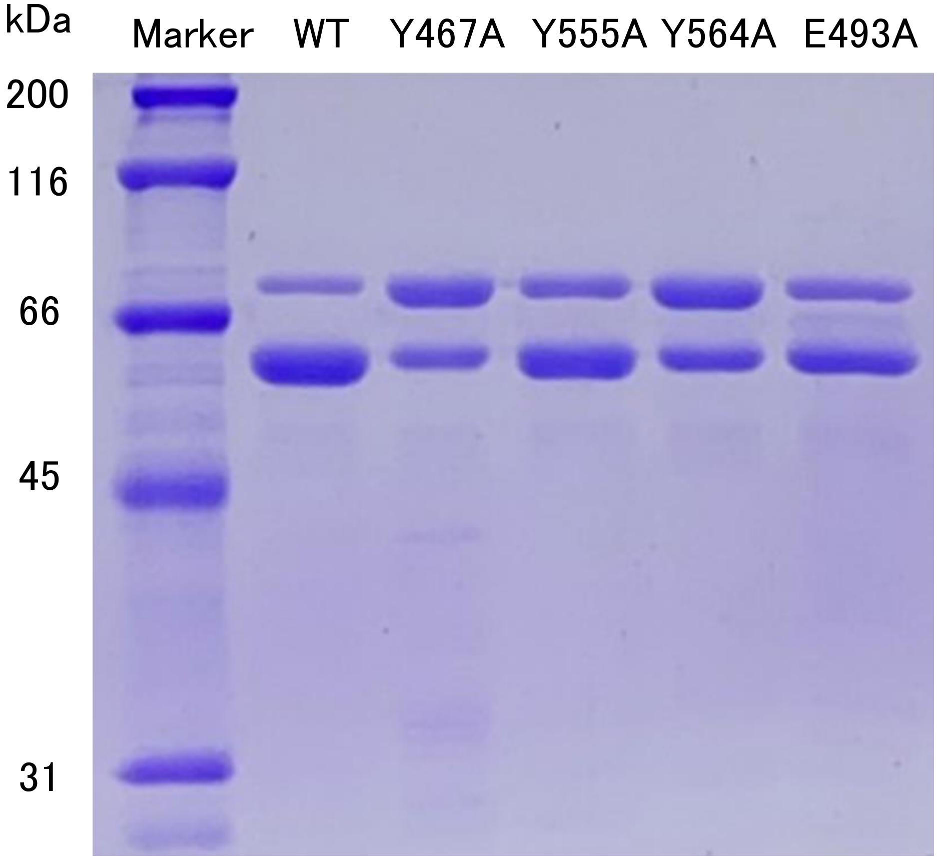

Fig. S8. SDS-PAGE of the wild-type Ghcol (WT) and its variants,. One μg of protein was applied to 12.5% acrylamide gel.

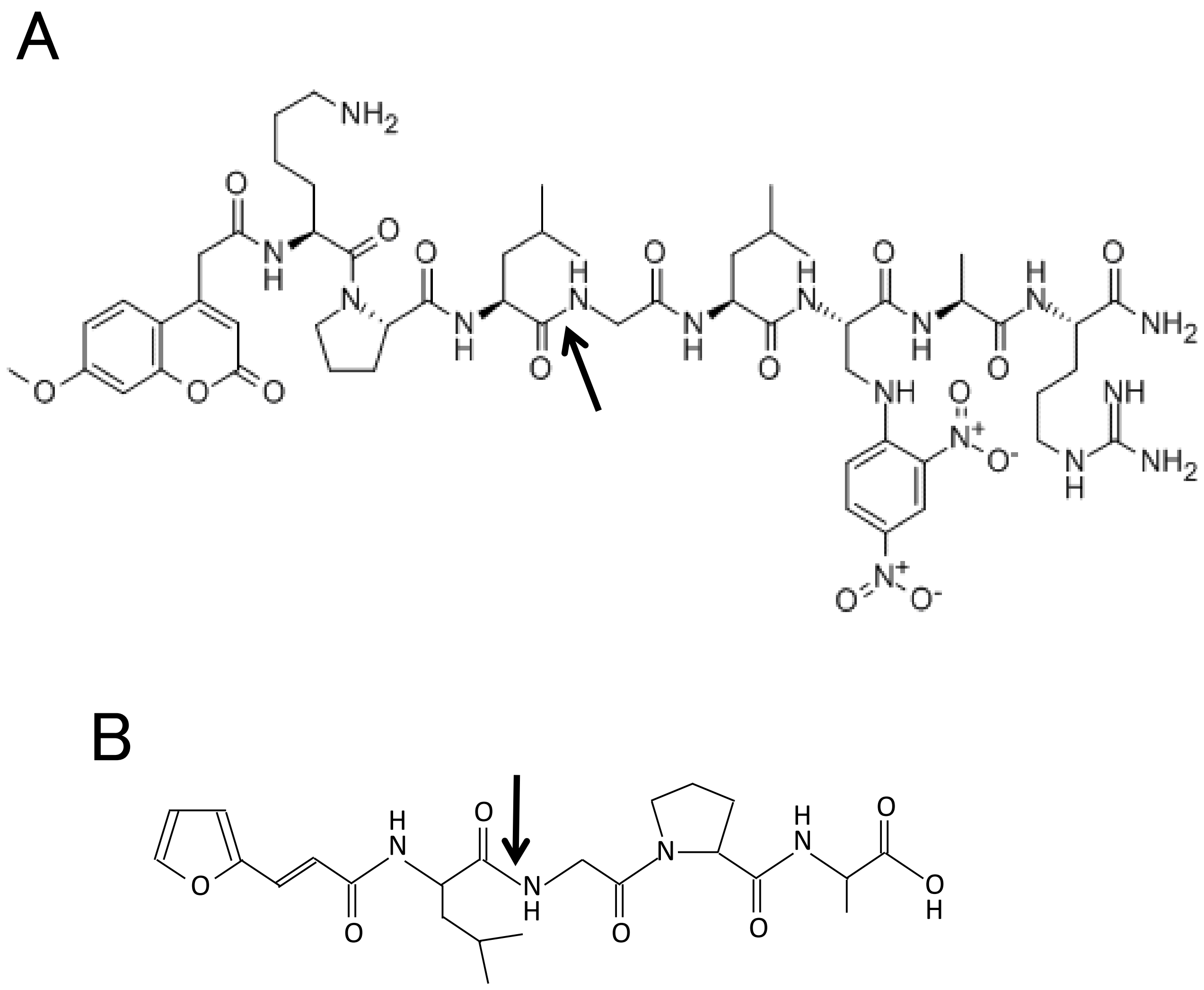

Fig. S9. Structure of synthetic substrates. (A) MOCAc-KPLGL(Dpa)-AR. (B) FALGPA. The arrow indicates the cleavage site by Ghcol, ColH, and ColG.

A

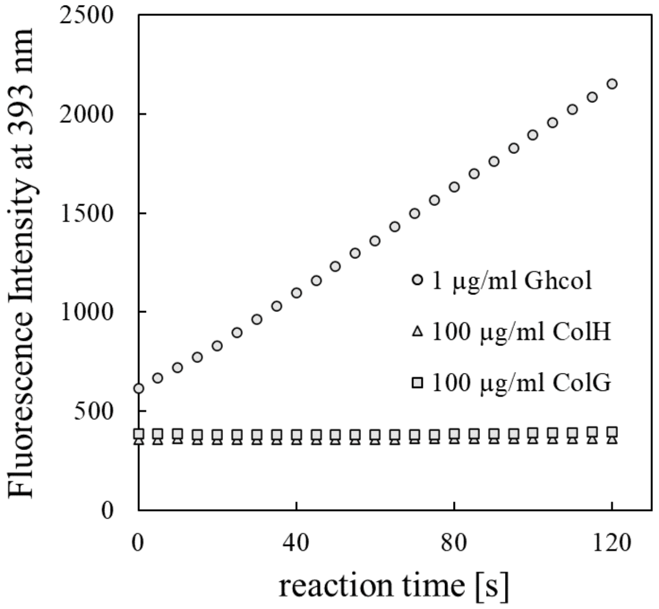

B

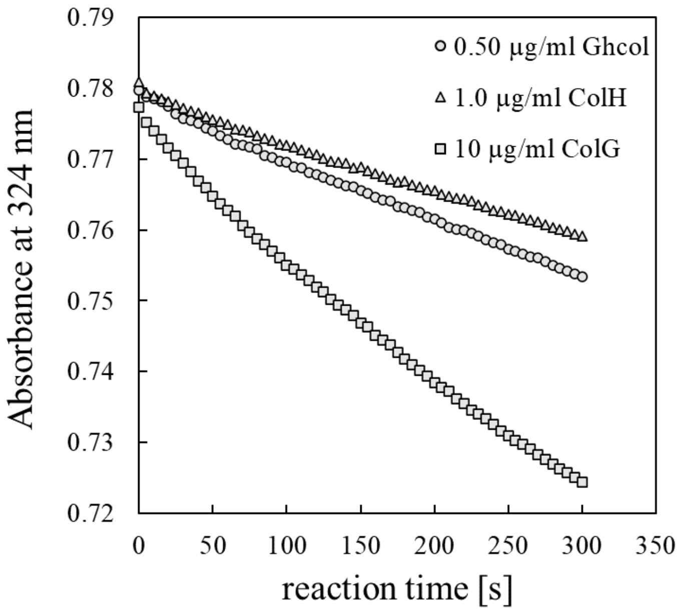

Fig. S10. Hydrolysis of synthetic peptides. (A) MOCAc-KPLGL(Dpa)-AR peptide. The reaction was carried out in 5 mM HEPES-NaOH buffer (pH 7.0) with 1.0 μg/mL Ghcol, 100 μg/mL ColH, or 100 μg/mL ColG in the presence of 1.0 μM MOCAc-KPLGL(Dpa)-ARat 25°C. (D) Hydrolysis of FALGPA. The reaction was carried out in 100 mM HEPES-NaOH buffer (pH 7.5), 200 mM NaCl, 10 mM $CaCl_2$ 10 μM $ZnCl_2$ with 0.50 μg/mL Ghcol, 1.0 μg/mL ColH, or 10 μg/mL ColG in the presence of 80 μM FALGPA at 25°C. One of the representative data is shown.
